# ﻿The genus *Euphitrea* Baly, 1875 (Coleoptera, Chrysomelidae, Galerucinae, Alticitae) in Taiwan, with description of three new species

**DOI:** 10.3897/zookeys.1252.150362

**Published:** 2025-09-19

**Authors:** Chi-Feng Lee

**Affiliations:** 1 Applied Zoology Division, Taiwan Agricultural Research Institute, Taichung 413, Taiwan Taiwan Agricultural Research Institute Taichung Taiwan

**Keywords:** Asteraceae, Begoniaceae, Cannabaceae, Lythraceae, Moraceae, Polygonaceae, Ulmaceae, Urticaceae

## Abstract

Three previously described species of *Euphitrea* from Taiwan are recognized and redescribed: *E. flavicornis* (Chen, 1933), *E. nisotroides* (Chen, 1933), and *E. taiwana* Kimoto, 1991. Additionally, three new species from Taiwan are described: *E. tsoui***sp. nov.**, *E. houjayi***sp. nov.**, and *E. jungchani***sp. nov.** The species descriptions include illustrations of aedeagi, antennae, gonocoxae, abdominal ventrite VIII, and spermathecae. Lectotypes are designated for *Neorthaea flavicornis* Chen, 1933 and *N. nisotroides* Chen, 1933.

## ﻿Introduction

The generic boundaries of *Euphitrea* Baly have long been problematic. Some members of the genus could not be identified by using existing keys due to their diverse external morphologies and body shapes, and the genus was redefined by Beenen (2015) based on a combination of the following characters: appendiculate claws, small grooves at the base of the pronotum, only hind tibia with a spine, metasternum covering the apex of the channeled prosternum, second antennomere slightly shorter than third, and presence of furrows lateral to the vertex.

Forty-seven species of *Euphitrea* are described from the Oriental region (Wang et al. 2008; Medvedev 2009; Döberl 2011; Beenen 2015). Taiwanese species are characterized by their small sizes (< 4.0 mm in length), flat and oval body shapes, and longitudinally convex vertices. Only three species have been described from Taiwan: *E. flavicornis* (Chen, 1933), *E. nisotroides* (Chen, 1933), and *E. taiwana* Kimoto, 1991. Adults are common in various forest types and easily collected by sweeping (Lee et al. 2017). More than 2,200 specimens were available for study, including historical collections at several museums, and extensive collections made by the Taiwan Chrysomelid Research Institute, and captured using Malaise traps set up at Tahanshan (大漢山) and Lichia (利嘉). The true species diversity and distributions can now be presented based on sufficient material.

## ﻿Materials and methods

For taxonomic study, abdomens of adults were separated from the forebodies and boiled in 10% KOH solution, followed by washing in distilled water to prepare genitalia for illustrations. The genitalia were then dissected from the abdomens, mounted on slides in glycerin, and studied and drawn using a Leica M165 stereomicroscope. For detailed examinations, a Nikon ECLIPSE 50i microscope was used.

At least three males and females from each species were examined to delimit variability of diagnostic characters. For species collected from more than one locality or with color variations, at least one pair of each sex from each locality and color morph was examined. Length was measured from the anterior margin of the eye to the elytral apex, and width at the greatest width of the elytra. Nomenclature for morphological structures of adults follows Duckett and Daza (2004). Names of plant species follows the Taiwan Encyclopedia of Life (2024), TaiEOL.

Specimens studied herein are deposited at the following institutes and collections:

**KMNH**Kitakyushu Museum of Natural History and Human History, Kitakyushu, Japan [Yûsuke Minoshima];

**NHMUK**The Natural History Museum, London, UK [Michael F. Geiser];

**NMNS**National Museum of Natural Science, Taichung, Taiwan [Bao-Cheng Lai];

**SDEI** Senckenberg Deutsches Enomologisches Institut, Müncheberg, Germany [Mandy Shröter];

**TARI**Applied Zoology Division, Taiwan Agricultural Research Insitut, Taichung, Taiwan [Chi-Feng Lee].

## ﻿Results

### ﻿*Euphitrea flavicornis* species group

Adults of this group can be distinguished from those of the *E. nisotroides* group by the dark metallic bronze bodies, and yellow antennae (reddish brown prothoraxes and heads, dark brown antennae except several basal antennomeres in *E. nisotroides* group), slightly swollen and asymmetrical tarsi I in males (strongly swollen and symmetrical tarsi I of males in *E. nisotroides* group), ventral surface of aedeagus without membranous areas (ventral surface of aedeagus with membranous areas in *E. nisotroides* group); and frontal ridge flat (frontal ridge convex in *E. nisotroides* group).

#### ﻿Included species

*Euphitrea flavicornis* (Chen, 1933), *E. houjayi* sp. nov., *E. jungchani* sp. nov., and *E. tsoui* sp. nov. Assignment of other species to this species group (or other groups) need further studies.

##### ﻿ Euphitrea flavicornis

Taxon classificationAnimaliaColeopteraChrysomelidae

(Chen, 1933)

96E2102F-A40F-5845-AF47-BC665B47C7D8

**Figure 1.**
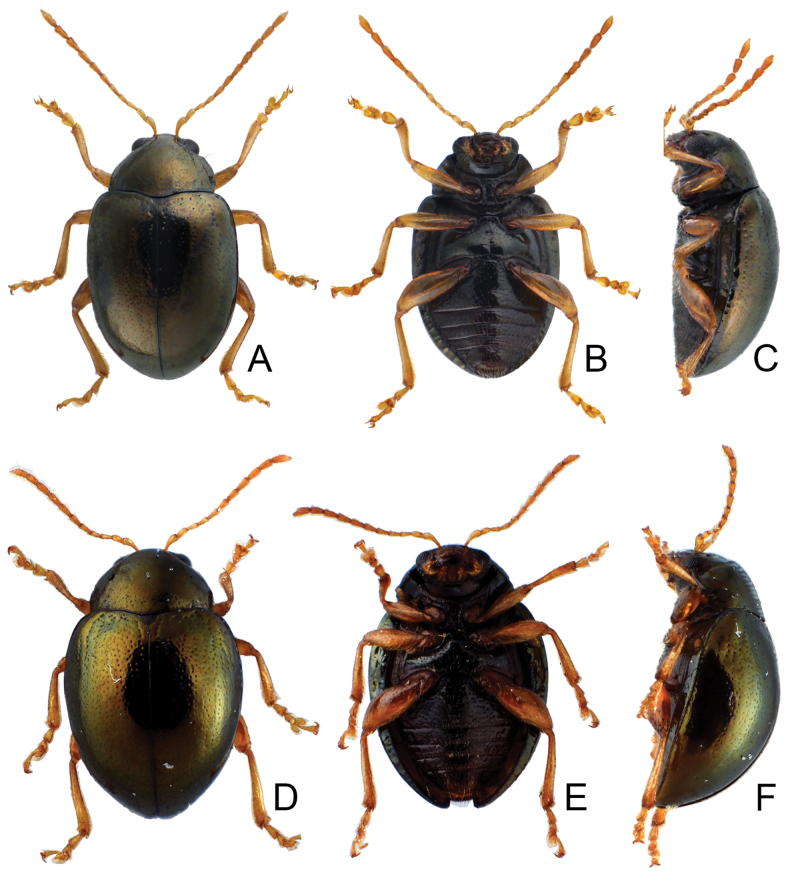
Habitus of *Euphitrea* species. **A***E. flavicornis* (Chen), male, dorsal view **B** ditto, ventral view **C** ditto, lateral view **D***E. houjayi* sp. nov., female, dorsal view **E** ditto, ventral view **F** ditto, lateral view.

**Figure 2.**
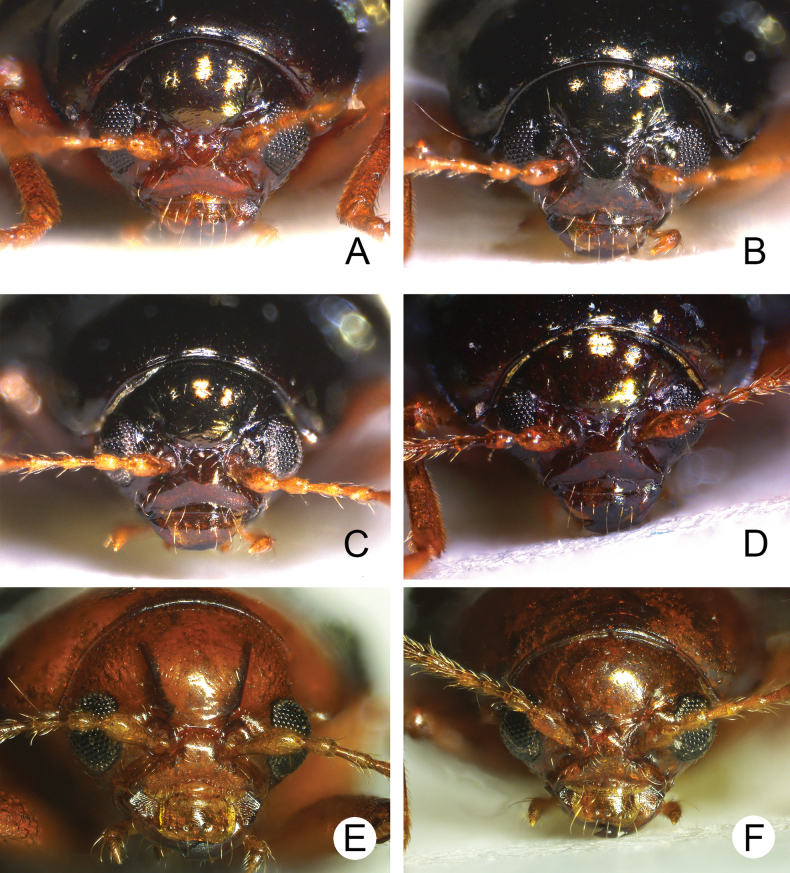
Head, front view. **A***Euphitrea flavicornis* (Chen) **B***A. houjayi* sp. nov. **C***A. jungchani* sp. nov. **D***A. tsoui* sp. nov. **E***A. nisotroides* (Chen) **F***A. taiwana* Kimoto.

**Figure 3.**
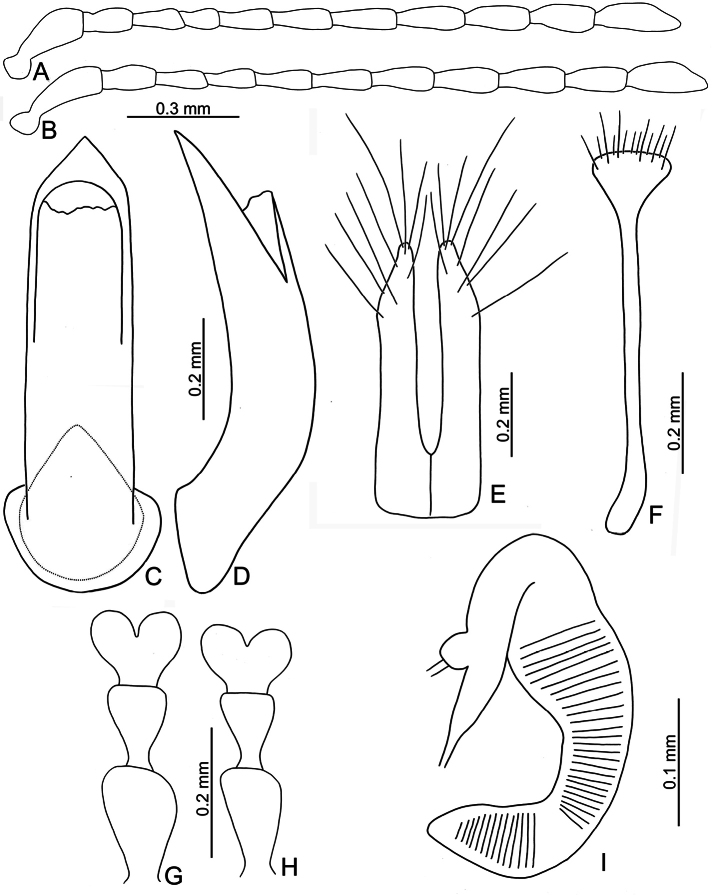
*Euphitrea flavicornis* (Chen) **A** antenna, male **B** antenna, female **C** aedeagus, dorsal view **D** aedeagus, lateral view **E** gonocoxae **F** abdominal ventrite VIII, female **G** tarsus, male **H** tarsus, female **I** spermatheca.

**Figure 4.**
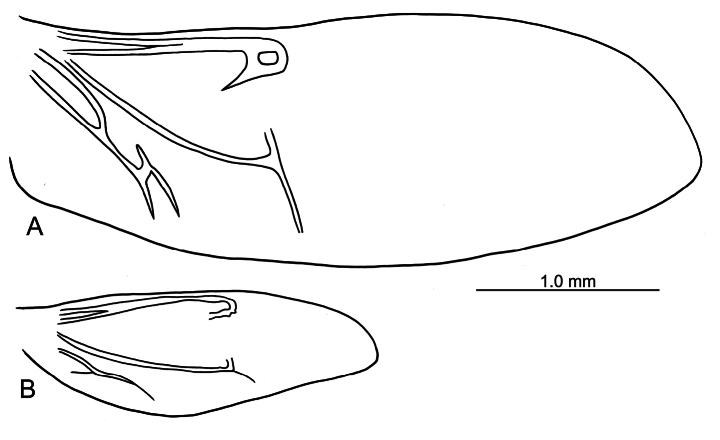
Hind wing **A***E. flavicornis* (Chen) **B***E. houjayi* sp. nov.

**Figure 5.**
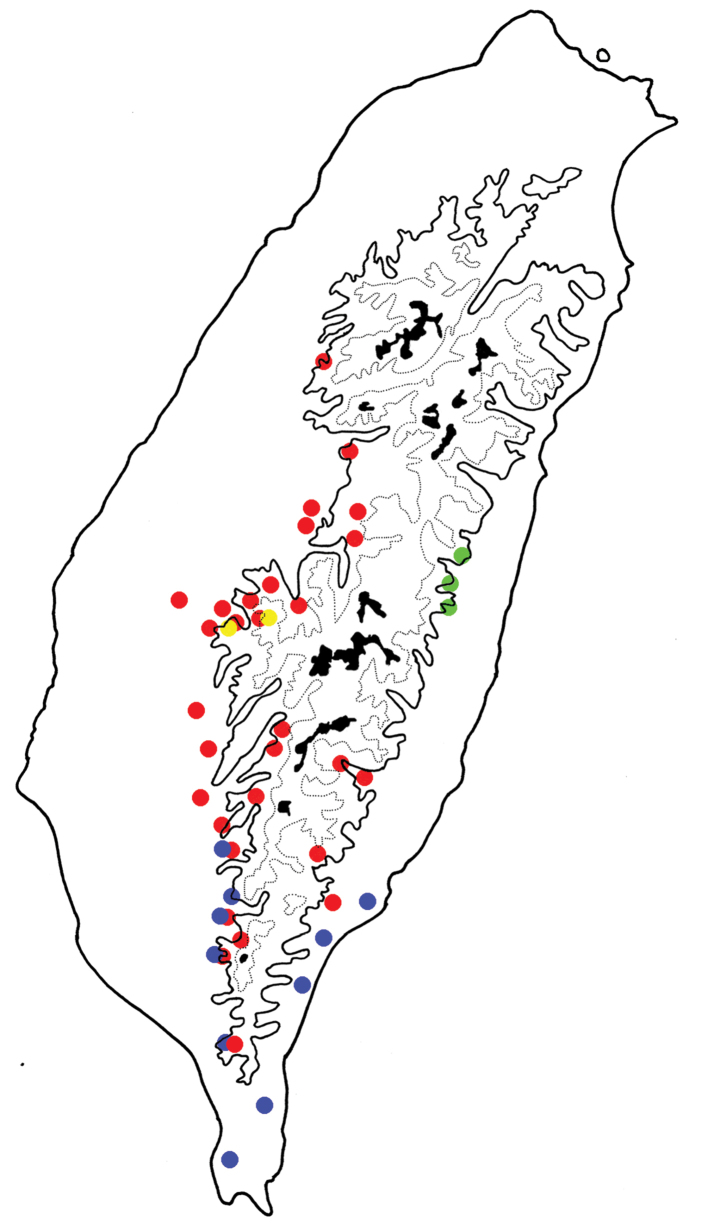
Distribution map of *Euphitrea flavicornis* species group in Taiwan; solid line 1000 m, broken line 2000 m, black areas 3000 m. Red dots *E. flavicornis* (Chen), blue dots *E. tsoui* sp. nov., *g*reen dots *E. jungchani* sp. nov., yellow dots *E. houjayi* sp. nov.

Figs 1A–C, 2A, 3, 4A, 5

Neorthaea flavicornis Chen, 1933: 90 (Taiwan: Taihorin); Chûjô 1935: 473 (part); Chûjô 1963: 399 (faunistic records); Chûjô 1965: 102 (faunistic records); Kimoto 1966: 35 (faunistic records); Kimoto 1970: 215 (faunistic records).
Euphitrea flavicornis : Kimoto 1986: 59 (faunistic records); Kimoto 1989: 263 (faunistic records); Kimoto 1991b: 19 (faunistic records); Bezděk and Konstantinov 2024: 509 (catalogue).

###### Type material examined.

***Lectotype*** • ♀ (SDEI, here designated to clarify its taxonomic status), labeled: “Taihorin (= Talin, 大林) / Formosa / H. Sauter, 1911 [p, w] // 7.VIII. [p, w] // Chen det [h, w] // Syntypus [p, r] // Neorthaea / flavicornis / Chen [h, w] // SDEI Müncheberg / Col – 19911 [p, g]”. ***Paralectotypes*** • Five types bearing the same labels are deposited at the SDEI (Chen 1933).

###### Additional material examined.

**Taiwan. Chiayi** • 2♀ (originally pinned with the same pin, TARI), Arisan (= Alishan, 阿里山), 10.X.1912, leg. I. Nitobe • 1♂ (TARI), same locality, 27.V.1917, leg. T. Shiraki • 5♀ (2♀: TARI; 3♀: NHMUK), same locality, 2–23.X.1918, leg. J. Sonan • 1♀ (TARI), same locality, 24.X.1933, leg. M. Chujo • 1♂, 1♀ (TARI), Funkiko (= Fenchifu, 奮起湖), 29.IV.1931, leg. T. Shiraki • 1♀ (TARI), same locality, 9.VIII.1940, leg. K. Endo • 3♂, 4♀ (KMNH), same locality, 8.VIII.1990, leg. S. Kimoto • 4♂, 1♀ (TARI), Juitai trail (瑞太古道), 15.IX.2013, leg. W.-C. Liao • 1♂, 2♀ (TARI), Laichitashan (來吉塔山), 19.III.2009, leg. H. Lee • 6♂, 10♀ (SDEI), Suisharyo (= Shuisheliao, 水社寮), X.1911, leg. H. Sauter • 1♂ (TARI), Tutzuhu (杜仔湖), 14.III.2015, leg. W.-C. Liao; **Kaohsiung** • 6♂, 1♀ (TARI), Chungchihkuan (中之關), 16.IV.2012, leg. L.-P. Hsu • 1♀ (TARI), same but with “15.IV.–10.X.2012” • 2♂, 3♀ (TARI), same but with “10–13.X.2012” • 3♂, 3♀ (TARI), Erhchituan (二集團), 8.III.2013, leg. B.-X. Guo • 1♂ (SDEI), Kosempo (= Chiasien, 甲仙), 22.V.1912, leg. H. Sauter • 2♂, 1♀ (TARI), Likuan (禮觀), 30.IV.2023, leg. Y.-F. Hsu • 4♂, 2♀ (KMNH), Meishan-Tinchi (梅山-天池), 29.VI.1986, leg. K. Baba • 1♂, 10♀ (KMNH), Shaping (扇平), 5.V.1986, leg. K. Baba • 1♂ (TARI), Tengchih (藤枝), 18.II.2007, leg. S.-F. Yu • 8♂, 1♀ (TARI), same locality, 2–5.VI.2008, leg. C.-F. Lee • 2♂ (TARI), same but with “26.V.2009” • 5♂, 5♀ (TARI), same locality, 31.VII.2008, leg. C.-T. Yao • 1♂ (TARI), 7.IX.2012, leg. W.-C. Liao • 1♀ (TARI), same but with “31.III.2013” • 1♀ (TARI), same but with “13.IV.2013” • 1♂, 2♀ (TARI), same locality, 18.IV.2013, leg. Y.-T. Chung • 1♀ (TARI), Tona trail (多納林道), 3.II.2013, leg. B.-X. Guo; 1♂, 2♀ (TARI), same locality, 3.II.2013, leg. W.-C. Liao • 6♂, 1♀ (TARI), same locality, 25.II.2013, leg. Y.-T. Chung; **Nantou** • 1♀ (TARI), Ailan (愛蘭), 30.IV.2009, leg. W.-T. Liu • 1♂, 1♀ (TARI), Choshe (卓社), 8.VII.2017, leg. B.-H. Kuo • 1♂ (TARI), Fenghuangshan (鳳凰山), 8.VI.2013, Y.-T. Wang • 1♀ (KMNH), Hsitou (溪頭), 31.III.1980, leg. K. Sugiyama • 1♀ (TARI), Huisun Forest Recreation (惠蓀林場), 23.IV.2015, leg. Y.-T. Chung • 8♂, 6♀ (TARI), Lienhuachi (蓮華池), 23–26.V.1980, leg. K. S. Lin & B. H. Chen • 1♀ (TARI), same locality, 4.IV.2016, leg. H.-T. Shih • 2♂, 1♀ (TARI), Tungpu (東埔), 28.IV.–2.V.1981, leg. T. Lin & C. J. Lee • 1♂, 1♀ (TARI), same locality, 18–23.XI.1981, leg. T. Lin & W. S. Tang • 12♂, 14♀ (TARI), same locality, 19–23.VII.1982, leg. L. Y. Chou & T. Lin • 17♂, 7♀ (TARI), same locality, 20–24.VI.1983, leg. K. C. Chou & C. Y. Wong • 1♂ (TARI), same locality, 16–20.IV.1984, leg. K. C. Chou & C. H. Yung • 1♀ (TARI), 8.I.2009, leg. H. Lee • 2♂, 1♀ (TARI), Hsitou (溪頭), 25.XII.2008, leg. C.-F. Lee • 2♀ (TARI), Wanfengtsun (萬豐村), 13.IV.2010, W.-T. Liu; **Pingtung** • 1♀ (TARI), Jinshuiying (浸水營), 12.IV.2012, leg. C.-F. Lee • 1♀ (TARI), same locality, 2.V.2020, leg. F.-S. Hu • 4♂, 10♀ (TARI), Machia (瑪家), 11.III.2013, leg. Y.-T. Chung • 1♀ (TARI), same but with “12.III.2014” • 1♂ (TARI), Peitawushan (北大武山), 5.II.2011, leg. J.-C. Chen • 1♂, 1♀ (TARI), same locality, 6.VIII.2016, leg. Y.-T. Chung • 1♂, 1♀ (TARI), Tahanshan (大漢山), 22.II.2007, leg. S.-F. Yu • 1♂, 1♀ (TARI), same but with “4.VII.2008” • 5♂, 6♀ (TARI), same locality, 6.II.2008, leg. M.-H. Tsou; 5♀ (TARI), same locality, 8.V.2009, leg. U. Ong • 1♀ (TARI), same locality, 10.IV.2012, leg. J.-C. Chen • 1♂ (TARI), same locality, 19.VII.2012, leg. C.-F. Lee • 3♀ (TARI), same locality, Malaise trap, 15.III.–3.IV.2020, leg. Y.-C. Chiu • 1♂, 1♀ (TARI), same but with “3.IV.–2.V.2020” • 6♂, 11♀ (TARI), same but with “2.V.–6.VI.2020” • 1♂, 1♀ (TARI), same but with “6–25.VI.2020” • 4♂, 3♀ (TARI), same but with “25.VI.–24.VII.2020” • 6♂, 3♀ (TARI), same but with “24.VII.–29.VIII.2020” • 6♂, 2♀ (TARI), same but with “29.VIII.–19.IX.2020” • 3♂ (TARI), same but with “19.IX.–24.X.2020” • 1♀ (TARI), same but with “24.X.21XI.2020” • 1♀ (TARI), Taiwu (泰武), 14.IX.2017, leg. Y.-T. Chung; **Taichung** • 1♂, 1♀ (TARI), Wushihkeng (烏石坑), 13.VII.2008, leg. M.-H. Tsou; **Tainan** • 2♂, 1♀ (TARI), Kantoushan (崁頭山), 14.III.2010, leg. M.-H. Tsou • 1♂ (TARI), Meiling (梅嶺), 12.III.2011, leg. M.-L. Jeng; **Taitung**: 1♂ (TARI), Lichia (利嘉), 2.VI.2009, leg. U. Ong • 2♂, 4♀ (TARI), same locality, 15.VII.2014, leg. Y.-T. Chung • 12♂, 20♀ (TARI), same locality, 16–17.VII.2014, leg. Y.-T. Wang • 2♂, 2♀ (TARI), same locality, 9.IV.2016, leg. S.-P. Wu • 1♀ (TARI), same but with “5.VII.2017” • 1♂, 2♀ (TARI), same locality, 29.II.–22.III.2020, Malaise trap, leg. Y.-C. Chiu • 2♂, 2♀ (TARI), same but with “19.IV.–30.V.2020” • 1♂ (TARI), same but with “30.V.–26.VI.2020” • 1♀ (TARI), same but with “26.VI.–23.VII” • 2♂, 1♀ (TARI), same but with “23.VII.–28.VIII.2020” • 2♂, 2♀ (TARI), Liyuan (栗園), 23.VI.2010, leg. M.-H. Tsou • 1♀ (TARI), same locality, 19.VI.2013, leg. C.-F. Lee • 2♂ (TARI), same locality, 28.III.2014, leg. W.-C. Huang • 1♂, 1♀ (TARI), Motien (摩天), 23.V.2011, leg. C.-F. Lee • 7♂, 8♀ (TARI), Yanping trail (延平林道), 5.III.2016, leg. S.-P. Wu.

###### Redescription.

***Adults*.** Length 2.7–3.4 mm, width 1.7–2.1 mm (*n* = 387). General color dark metallic bronze (Fig. 1A–C); antennae, legs, and mouthparts yellow. Frontal ridge flat. Furrows lateral to vertex shallow and short, apically reaching or slightly exceeding apical margins of eyes (Fig. 2A). Antennae (Fig. 3A) filiform in males, ratios of lengths of antennomeres I–XI 1.0: 0.5: 0.5: 0.4: 0.6: 0.7: 0.7: 0.8: 0.8: 0.7: 1.0; ratios of length to width from antennomeres I–XI 3.1: 2.0: 2.3: 1.9: 2.6: 2.8: 3.0: 2.8: 2.8: 2.4: 3.2; similar in females, ratios of lengths of antennomeres I–XI (Fig. 3B) 1.0: 0.5: 0.5: 0.5: 0.6: 0.6: 0.6: 0.7: 0.7: 0.6: 0.8; ratios of length to width from antennomeres I–XI 3.5: 2.1: 2.7: 2.6: 2.9: 2.9: 2.7: 2.6: 2.7: 2.5: 2.9. Pronotum 1.6–1.8× wider than long; disc shining, with dense, coarse punctures, slightly convex; lateral margins rounded; apical margins slightly concave; basal margin medially convex. Elytra 1.2–1.3× longer than wide; disc with coarse punctures arranged into paired longitudinal lines, with fine punctures between coarse punctures; lateral margins rounded, widest at basal 1/4; humeral calli well developed, hind wings normal (Fig. 4A). Tarsomeres I of front and middle legs slightly swollen and asymmetrical in males (Fig. 3G); normal in females (Fig. 3H). Apical margin of abdominal ventrite V in males trilobed, notches shallow; apical margin of abdominal ventrite V broadly rounded in females. Aedeagus (Fig. 3C, D) narrow, ~ 4.5× longer than wide; parallel-sided, moderately apically narrowed at apical 1/7, apex pointed; moderately curved in lateral view; tectum membranous. Endophallic spiculae reduced. Gonocoxae (Fig. 3E) longitudinal and connected at base; gonocoxa approximate, apically narrow; apex narrowly rounded or truncate; with eight long apical setae. Ventrite VIII (Fig. 3F) well sclerotized and small, several short setae arranged into transverse line along apical margin, apical margin rounded, with three long setae at sides, spiculum extremely long. Spermathecal receptaculum (Fig. 3I) moderately swollen; pump wide and curved, with long apical process; sclerotized spermathecal duct short beyond spermathecal gland, base of spermathecal gland enlarged and sclerotized.

###### Diagnosis.

Adults of *Euphitrea flavicornis* are similar to those of *E. jungchani* sp. nov. with normal humeral calli on elytra (reduced in *E. houjayi* sp. nov.) and oval bodies and elytra 1.2–1.3 longer than wide (transversely oval bodies and elytra 1.1× longer than wide in *E. tsoui* sp. nov.). In addition to the allopatric distributions of both species, some genital structures are diagnostic, including straight apices of aedeagi of *E. flavicornis* in lateral view (Fig. 3D) and membranous tectum (Fig. 3C) (aedeagi of *E. jungchani* sp. nov. subapically curved in lateral view (Fig. 8D) and sclerotized tectum (Fig. 8C)), swollen spermathecal receptaculum (Fig. 3I) (not swollen in *E. jungchani* sp. nov.; Fig. 8I), and comparatively narrower apices of abdominal ventrite VIII in females (Fig. 3F) (comparatively wider apices of abdominal ventrite VIII in females of *E. jungchani* sp. nov.; Fig. 8F).

###### Food plants.

Urticaceae: Elatostema lineolatum var. majus Wedd.; *Pilea melastomoides* (Poir.) Wedd.; Begoniaceae: *Begonia tengchiana* C.I. Peng & Y.K. Chen.

###### Biological notes.

Lee et al. (2017) presented results of a project on species diversity of leaf beetles at the Xitou Nature Education Area (溪頭教育園區) in Central Taiwan. They collected leaf beetles by sweeping one day every month from November 2014 to June 2016. *Euphitrea flavicornis* was one of the dominant species, with 781 adults represented in collections during the study. Adults were present during all years, although few were collected during winter. Similar results were documented in populations collected using Malaise traps set at Tahanshan (大漢山), South Taiwan (see “Additional material examined”).

###### Distribution.

Central and south Taiwan (Fig. 5). Whether populations of this species can survive at Mt. Alishan, more than 2000 m elevation, is unknown. Specimens collected from Mt. Alishan are more than 100 years old. The word “Arisan (= Alishan)” during the past may refer to a much wider range than at present, indicating the whole township. Thus, these old specimens may not have been collected at high altitudes.

##### ﻿ Euphitrea houjayi sp. nov.

Taxon classificationAnimaliaColeopteraChrysomelidae

E4DE73DA-42F5-5640-BDCA-01938001C8C9

https://zoobank.org/846DC527-86CC-4D37-B2C8-2762B7ECE6BB

**Figure 6.**
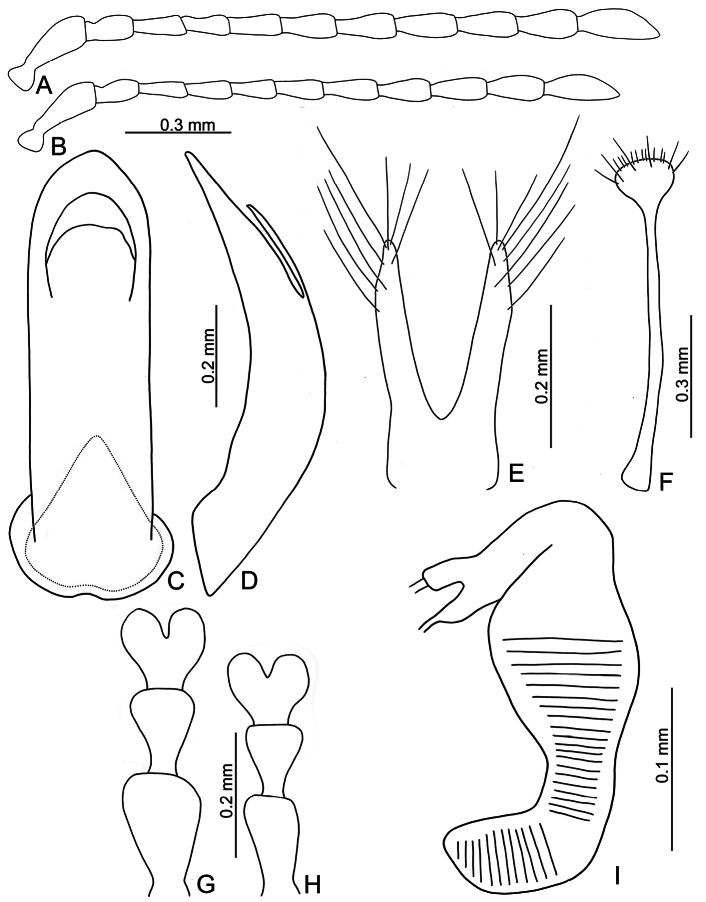
*Euphitrea houjayi* sp. nov. **A** antenna, male **B** antenna, female **C** aedeagus, dorsal view **D** aedeagus, lateral view **E** gonocoxae **F** abdominal ventrite VIII, female **G** tarsus, male **H** tarsus, female **I** spermatheca.

Figs 1D–F, 2B, 4B, 5, 6

Neorthaea flavicornis : Chûjô 1935: 473 (part).

###### Type material.

***Holotype*** • ♂ (TARI): **Taiwan. Chiayi** • Alishan (阿里山), 25.IV.2009, leg. H.-J. Chen. ***Paratypes*** • 1♂, 3♀ (TARI), same data as holotype • 1♀ (TARI), same locality, 5–9.VIII.1981, leg. L. Y. Chou & S. C. Lin • 1♀ (TARI), same locality, 12.V.2011, leg. C.-F. Lee • 3♀ (TARI), same locality, 28.IX.2024, leg. Y.-C. Hsu • 1♂, 1♀ (originally pinned with the same pin, TARI), same locality (= Arisan), 10.X.1912, leg. I. Nitobe • 3♂, 1♀ (originally pinned with the same pin, TARI), same locality (= Arisan), VII. 1915, leg. M. Maki • 2♀ (TARI), same locality (= Arisan), 27.V.1917, leg. T. Shiraki • 9♂, 16♀ (KMNH), Fenchihu (奮起湖), 8.VIII.1990, leg. S. Kimoto. The specimens collected by Nitobe and Maki were misidentified as *E. flavicornis* by Chûjô (1935).

###### Description.

***Adults*.** Length 2.8–3.0 mm, width 1.7–1.9 mm (*n* = 43). General color blackish brown or dark reddish brown (Fig. 1D–F); antennae, legs, and mouthparts yellow; elytra dark metallic blue. Frontal ridge flat. Furrows lateral to vertex shallow and short, apically reaching or slightly exceeding apical margins of eyes (Fig. 2B). Antennae (Fig. 6A) filiform in males, ratios of lengths of antennomeres I–XI 1.0: 0.5: 0.6: 0.5: 0.6: 0.6: 0.7: 0.7: 0.7: 0.7: 0.9; ratios of length to width from antennomeres I–XI 2.7: 1.9: 2.5: 2.3: 2.5: 2.3: 2.4: 2.5: 2.4: 2.1: 2.8; similar in females, ratios of lengths of antennomeres I–XI (Fig. 6B) 1.0: 0.5: 0.5: 0.5: 0.5: 0.5: 0.6: 0.6: 0.6: 0.6: 0.9; ratios of length to width from antennomeres I–XI 3.1: 2.2: 3.2: 2.5: 2.5: 2.6: 2.4: 2.5: 2.5: 2.4: 3.0. Pronotum 1.7–1.8× wider than long; disc shining, with dense, coarse punctures, convex; lateral margins rounded; apical margins concave; basal margin medially convex. Elytra 1.2× longer than wide; disc with coarse punctures arranged into paired longitudinal lines, with fine punctures between coarse punctures; lateral margins strongly rounded, widest at basal 1/4; humeral calli reduced, hind wings reduced to 50% of normal wing size (Fig. 4B). Tarsomeres I of front and middle legs slightly swollen and asymmetrical in males (Fig. 6G); normal in females (Fig. 6H). Apical margin of abdominal ventrite V in males trilobed, notches shallow; apical margin of abdominal ventrite V broadly rounded in females. Aedeagus (Fig. 6C, D) wide, ~ 3.9× longer than wide; parallel-sided, moderately apically narrowed at apical 1/7, apex widely rounded; moderately curved in lateral view; tectum membranous. Endophallic spiculae reduced. Gonocoxae (Fig. 6E) longitudinal and connected at base; gonocoxa directed outwards, apically narrow; apex narrowly rounded or truncate; with eight long apical setae. Ventrite VIII (Fig. 6F) well sclerotized and small, several short setae arranged into transverse line along apical margin, apical margin rounded, with three long setae at sides, spiculum extremely long. Spermathecal receptaculum (Fig. 6I) strongly swollen; pump wide and curved; sclerotized spermathecal duct short beyond spermathecal gland, base of spermathecal gland enlarged and sclerotized.

###### Diagnosis.

Adults of *Euphitrea houjayi* sp. nov. are characterized by their reduced humeral calli on elytra and hind wings (normal humeral calli on elytra and hind wings in others). In addition, some genital characters are diagnostic, including wider aedeagi (Fig. 6C) (more slender aedeagi in *E. flavicornis* (Fig. 3C) and *E. jungchani* sp. nov. (Fig. 8C) and moderately swollen spermathecal receptaculum (Fig. 6I) (swollen spermathecal receptaculum in *E. jungchani* sp. nov. (Fig. 8I) and *E. tsoui* sp. nov. (Fig. 9I)).

###### Food plants.

Polygonaceae: *Persicaria thunbergii* (Siebold & Zucc.) H.Gross

###### Etymology.

This new species is named for Hou-Jay Chen (陳厚潔), the first person to collect specimens.

###### Distribution.

Only found at the Alishan (2200 m) and Fenchihu (1400 m) (Fig. 5). Current data indicate that only *Euphitrea houjayi* sp. nov. occurs at Alishan but not *E. flavicornis*. Interestingly, both species coexist at lower altitudes such as Fenchihu.

##### ﻿ Euphitrea jungchani sp. nov.

Taxon classificationAnimaliaColeopteraChrysomelidae

D882D705-6429-531F-8E64-23EF8A0A6177

https://zoobank.org/5012AC1B-53ED-4E78-AE3D-8A827618D87A

Figs 2C, 5, 7A–C, 8

###### Type material.

***Holotype*** • ♂ (TARI): **Taiwan. Hualien**: Juisui (瑞穗), 27.VI.2013, leg. Y.-T. Chung; ***Paratypes*** • 1♂ (TARI), same data as holotype • 4♂ (TARI), same locality, 17.IX.2024, leg. J.-C. Chen; 1♂ (NMNS), Fuyuan Forest Rec. Area (富源森林遊樂區), 27.V.1997, leg. C. W. & L. B. O’Brien • 1♂, 1♀ (TARI), Wanjung (萬榮), 11.VI.2011, leg. M.-H. Tsou • 4♀ (TARI), same locality (= Wanrong trail, 萬榮林道), 7.IX.2024, leg. J.-C. Chen • 2♂, 3♀ (TARI), same but with “16.IX.2024”.

###### Description.

**Figure 7.**
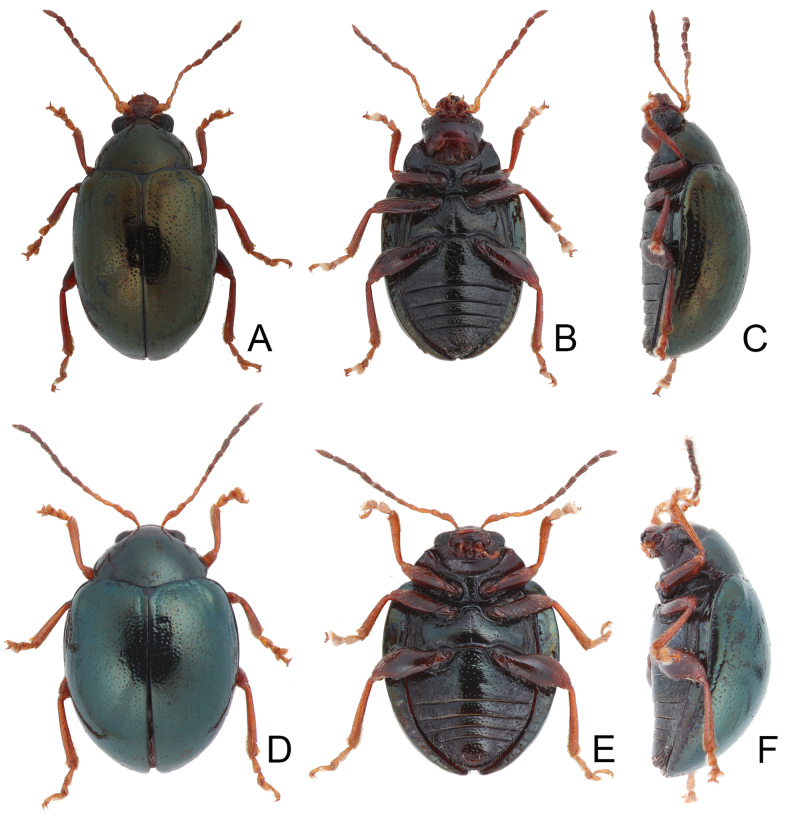
Habitus of *Euphitrea* species. **A***E. jungchani* sp. nov., female, dorsal view **B** ditto, ventral view **C** ditto, lateral view **D***E. tsoui* sp. nov., female, dorsal view **E** ditto, ventral view **F** ditto, lateral view.

**Figure 8.**
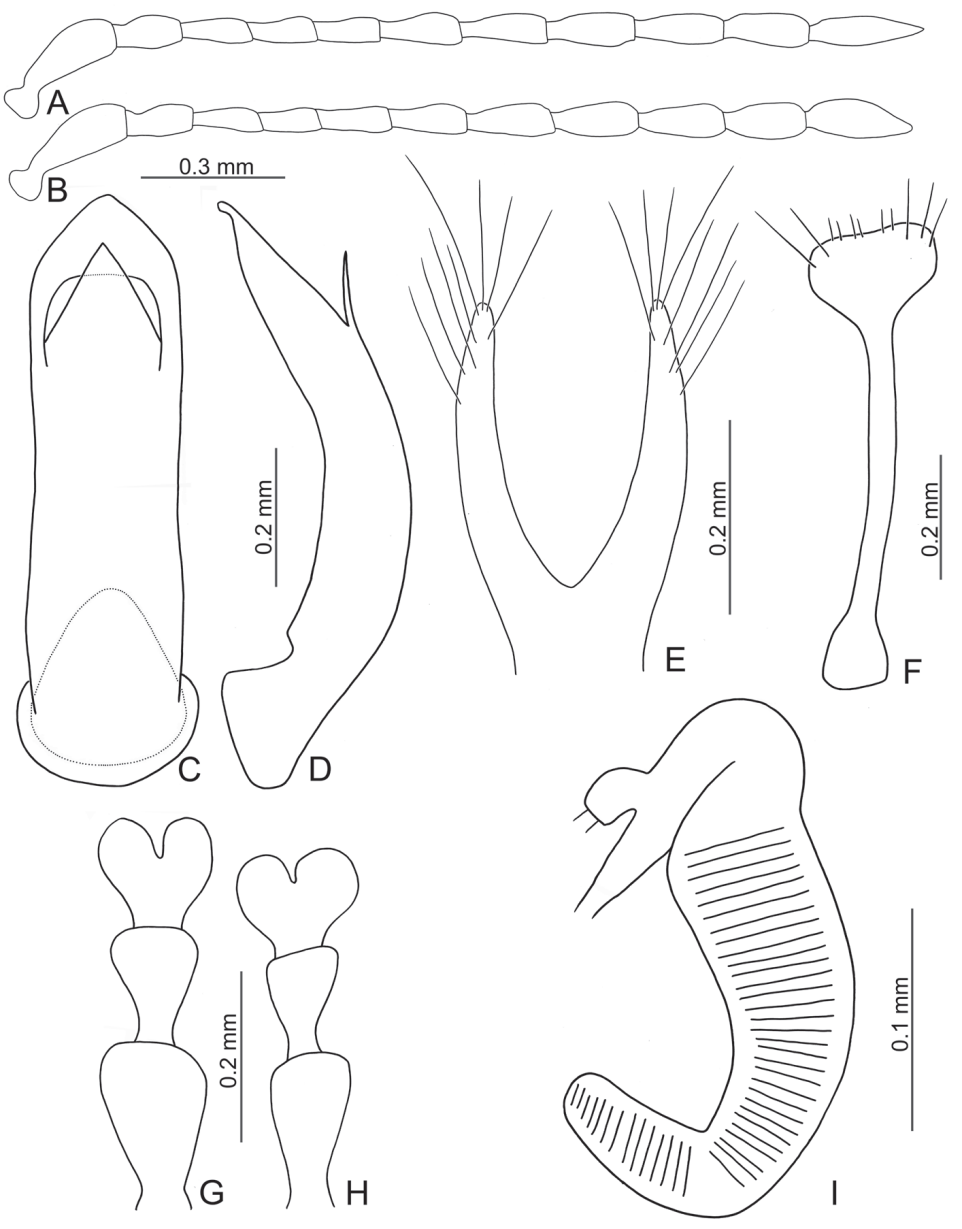
*Euphitrea jungchani* sp. nov. **A** antenna, male **B** antenna, female **C** aedeagus, dorsal view **D** aedeagus, lateral view **E** gonocoxae **F** abdominal ventrite VIII, female **G** tarsus, male **H** tarsus, female **I** spermatheca.

***Adults*.** Length 3.2–3.7 mm, width 1.8–2.1 mm (*n* = 18). General color dark metallic bronze (Fig. 7A–C); antennae, legs, and mouthparts yellow. Furrows lateral to vertex deep but short, apically slightly exceeding apical margins of eyes (Fig. 2C). Antennae (Fig. 8A) filiform in males, ratios of lengths of antennomeres I–XI 1.0: 0.5: 0.5: 0.5: 0.5: 0.6: 0.7: 0.7: 0.7: 0.7: 0.9; ratios of length to width from antennomeres I–XI 3.0: 2.1: 2.6: 2.4: 2.7: 2.5: 2.7: 2.7: 2.8: 2.6: 4.0; similar in females, ratios of lengths of antennomeres I–XI (Fig. 8B) 1.0: 0.5: 0.5: 0.4: 0.6: 0.6: 0.7: 0.7: 0.7: 0.6: 0.8; ratios of length to width from antennomeres I–XI 3.0: 2.2: 2.8: 2.3: 2.8: 2.6: 2.6: 2.6: 2.6: 2.3: 3.0. Pronotum 1.5–1.5× wider than long; disc shining, with dense, coarse punctures, convex; lateral margins rounded; apical margins concave; basal margin medially convex. Elytra 1.3–1.4× longer than wide; disc with coarse punctures arranged into paired longitudinal lines, with fine punctures between coarse punctures; lateral margins strongly rounded, widest at basal 1/3; humeral calli well developed, hind wings normal. Tarsomeres I of front and middle legs slightly swollen and asymmetrical in males (Fig. 8G); normal in females (Fig. 8H). Apical margin of abdominal ventrite V in males trilobed, notches shallow; apical margin of abdominal ventrite V broadly rounded in females. Aedeagus (Fig. 8C, D) wide, ~ 4.4× longer than wide; parallel-sided, moderately apically narrowed at apical 1/7, apex widely rounded; moderately curved in lateral view, and apex curved downwards; tectum sclerotized, apex pointed. Endophallic spiculae reduced. Gonocoxae (Fig. 8E) curved and connected at base; gonocoxa separated from each other, apically narrow; apex narrowly rounded or truncate; with eight long apical setae. Ventrite VIII (Fig. 8F) well sclerotized and several small, short setae arranged in a transverse line along apical margin, apical margin truncate but slightly concave at middle, with two or three long setae at sides, spiculum long. Spermathecal receptaculum (Fig. 8I) slightly swollen; pump wide and curved; sclerotized spermathecal duct short beyond spermathecal gland, base of spermathecal gland enlarged and sclerotized.

###### Diagnosis.

Adults of *Euphitrea jungchani* sp. nov. are similar to those of *E. flavicornis*, with normal humeral calli on elytra (reduced in *E. houjayi* sp. nov.), and oval bodies and elytra 1.2–1.3 longer than wide (transversely oval bodies and elytra 1.1× longer than wide in *E. tsoui* sp. nov.). In addition to the allopatric distributions of both species, some genital structures are diagnostic, including curved apices of aedeagi in lateral view (Fig. 8D) and sclerotized tectum (Fig. 8C) (aedeagi of *E. flavicornis* straight in lateral view (Fig. 3D) and membranous tectum (Fig. 3C)), slightly swollen spermathecal receptaculum (moderately swollen in *E. flavicornis*), and comparatively wider apices of abdominal ventrite VIII in females (Fig. 8I) (comparatively narrower apices of abdominal ventrite VIII in females of *E. flavicornis*, Fig. 3I).

###### Food plants.

Urticaceae: *Oreocnide pedunculata* (Shirai) Masam.

###### Etymology.

This new species is named for Jung-Chan Chen (陳榮章), the first person to collect specimens.

###### Distribution.

Eastern Taiwan. Specimens were collected from three localities in Hualien County, allopatric relative to the distribution of *E. flavicornis* (Fig. 5).

##### ﻿ Euphitrea tsoui sp. nov.

Taxon classificationAnimaliaColeopteraChrysomelidae

6D02A17B-634E-5079-84D6-8782E737B16B

https://zoobank.org/E6075035-BFB9-4620-89B4-5075AB6D0597

Figs 2D, 5, 7D–F, 9

Neorthaea flavicornis : Chûjô 1935: 473 (part).

###### Type material.

***Holotype*** • ♂ (TARI), **Taiwan. Taitung**: Imalintao (依麻林道), 4.II.2008, leg. M.-H. Tsao (= Tsou). ***Paratypes*** • 2♂, 12♀ (TARI), same data as holotype; **Kaohsiung** • 1♂ (TARI), Tonalintao (多納林道), 3.II.2013, leg. B.-X. Guo; **Pingtung** • 2♂, 1♀ (TARI), Koshun (= Henchun, 恆春), 25.IV.–25.V.1918, leg. J. Sonan • 1♀ (TARI), Machia (瑪家), 11.III.2013, leg. Y.-T. Chung • 5♂, 3♀ (TARI), same locality, 17.III.2013, leg. W.-C. Liao • 2♂, 4♀ (TARI), Shouka (壽卡), at Shihtzu township (獅子鄉), 10.XI.2012, leg. J.-C. Chen • 1♂, 3♀ (TARI), D(T)ahanshan (大漢山), 18.VII.2007, leg. C.-F. Lee • 1♂, 1♀ (TARI), same but with “26.III.2013” • 1♂ (TARI), same locality, 18.V.2009, leg. M.-L. Jeng • 3♂, 3♀ (TARI), same locality, 16.VI.2011, leg. J.-C. Chen • 4♀ (TARI), same but with “10.IV.2012” • 3♂, 1♀ (TARI), same locality, 14.VIII.2011, leg. Y.-T. Wang • 3♀ (TARI), same locality, 6.IV.2013, leg. W.-C. Liao • 1♂ (TARI), same but with “25.X.2014” • 2♂, 1♀ (TARI), same but with “13.VI.2015” • 1♂ (TARI), same locality, 4–5.VI.2013, leg. K. Takahashi • 1♂ (TARI), same locality, 29.VI.2013, leg. B.-X. Guo • 1♀ (TARI), same locality, 10.VII.2013, leg. Y.-T. Chung • 1♂, 1♀ (TARI), same but with “12.IX.2022” • 1♀ (TARI), same locality, Malaise trap, 15.III.–3.IV.2020, leg. Y.-C. Chiu • 1♂ (TARI), same but with “2–6.VI.2020” • 1♂, 1♀ (TARI), same but with “6.VI.–25.VI.2020” • 1♀ (TARI), same but with “25.VI.–24.VII.2020” • 1♀ (TARI), Taiwu (泰武), 27.IV.2023, leg. Y.-T. Chung • 1♀ (TARI), Wutai (霧台), 9.V.2009, leg. U. Ong; **Taitung** • 1♂, 2♀ (TARI), Chipen (知本), 17–18.II.1982, leg. L. Y. Chou & K. C. Chou • 1♂, 1♀ (TARI), same locality, 22.I.2013, leg. B.-X. Guo • 1♂, 1♀ (TARI), same locality, 23.I.2013, leg. Y.-T. Chung • 2♀ (TARI), Taimali (太麻里), 15.I.2009, leg. C.-F. Lee • 1♂, 1♀ (TARI), Taito (= Taitung, 台東), 25.II.–27.III.1919, leg. S. Inamura • 2♀ (NHMUK), same but with “leg. S. Inamura, J. Sonan, M. Yoshio”. Specimens collected by Sonan and Inamura were misidentified as *E. flavicornis* by Chûjô (1935).

###### Description.

**Figure 9.**
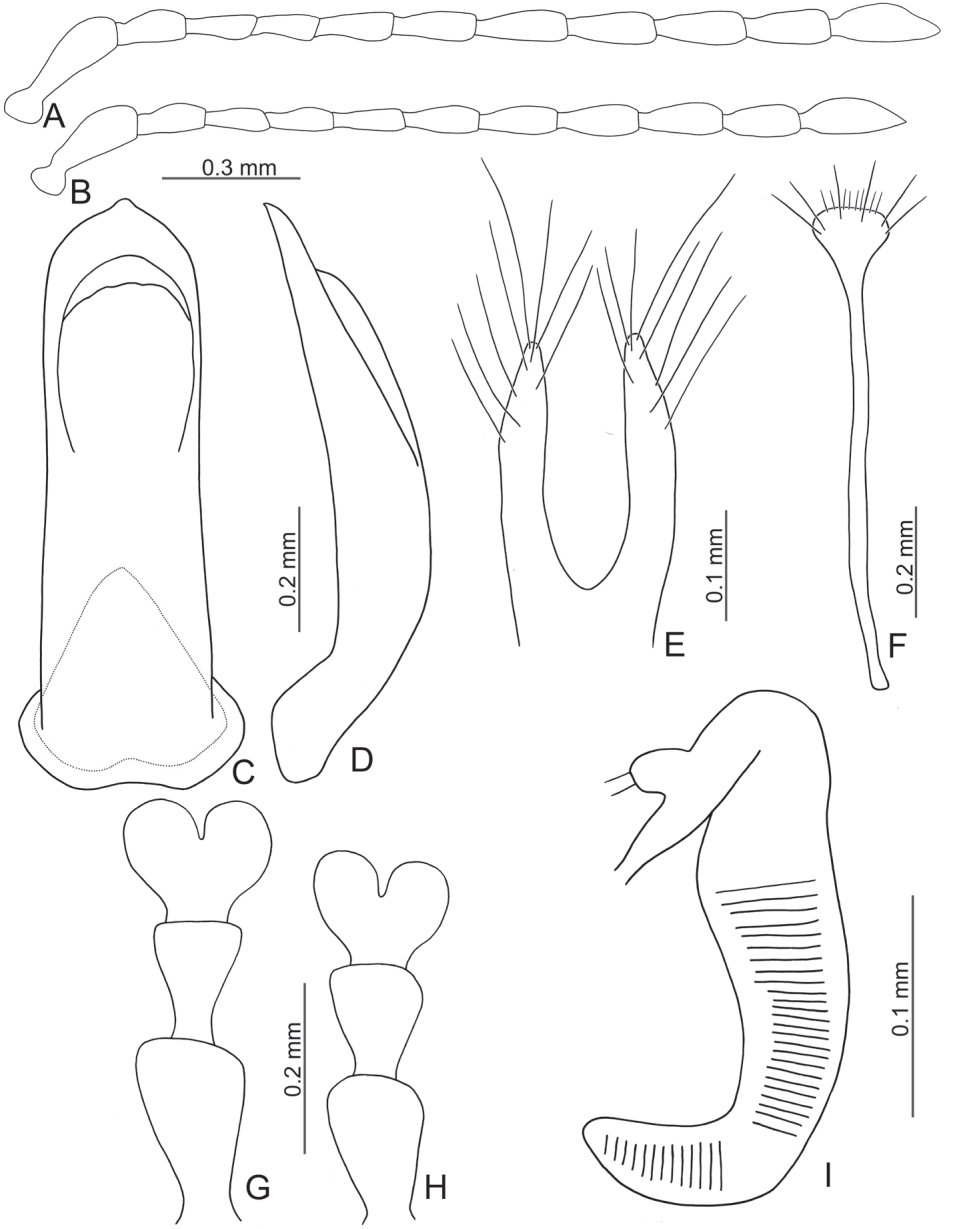
*Euphitrea tsoui* sp. nov. **A** antenna, male **B** antenna, female **C** aedeagus, dorsal view **D** aedeagus, lateral view **E** gonocoxae **F** abdominal ventrite VIII, female **G** tarsus, male **H** tarsus, female **I** spermatheca.

Length 3.2–3.5 mm, width 2.2–2.5 mm (*n* = 87). General color blackish brown or dark reddish brown (Fig. 7D–F); antennae, legs, and mouthparts yellow but antennae more or less darkened; elytra dark metallic blue. Frontal ridge flat. Furrows lateral to vertex shallow and short, apically reaching or slightly exceeding apical margins of eyes (Fig. 2D). Antennae (Fig. 9A) filiform in males, ratios of lengths of antennomeres I–XI 1.0: 0.5: 0.5: 0.4: 0.6: 0.6: 0.7: 0.7: 0.7: 0.6: 0.8; ratios of length to width from antennomeres I–XI 3.3: 2.1: 2.3: 2.5: 2.7: 2.9: 3.0: 2.8: 2.6: 2.4: 3.0; similar in females, ratios of lengths of antennomeres I–XI (Fig. 9B) 1.0: 0.6: 0.5: 0.6: 0.6: 0.6: 0.7: 0.7: 0.7: 0.6: 0.9; ratios of length to width from antennomeres I–XI 2.9: 2.4: 2.7: 2.6: 3.1: 2.8: 2.8: 2.6: 2.6: 2.4: 3.0. Pronotum 1.8–1.9× wider than long; disc shining, with dense, coarse punctures, convex; lateral margins rounded; apical margins concave; basal margin medially convex. Elytra 1.1× longer than wide; disc with coarse punctures arranged into paired longitudinal lines, with fine punctures between coarse punctures; lateral margins strongly rounded, widest at basal 1/4; humeral calli well developed, hind wings normal. Tarsomeres I of front and middle legs slightly swollen and asymmetrical in males (Fig. 9G); normal in females (Fig. 9H). Apical margin of abdominal ventrite V in males trilobed, notches shallow; apical margin of abdominal ventrite V broadly rounded in females. Aedeagus (Fig. 9C, D) wide, ~ 4.0× longer than wide; parallel-sided, moderately apically narrowed at apical 1/7, apex narrowly rounded; slightly curved in lateral view; tectum membranous. Endophallic spiculae reduced. Gonocoxae (Fig. 9E) longitudinal and connected at base; gonocoxa approximate, apically narrow; apex narrowly rounded or truncate; with eight long apical setae. Ventrite VIII (Fig. 9F) well sclerotized and small, several short setae arranged into transverse line along apical margin, apical margin rounded, with three long setae at sides, spiculum extremely long. Spermathecal receptaculum (Fig. 9I) slightly swollen; pump wide and curved; sclerotized spermathecal duct short beyond spermathecal gland, base of spermathecal gland enlarged and sclerotized.

###### Diagnosis.

Adults of *Euphitrea tsoui* sp. nov. are characterized by their transversely oval bodies with elytra 1.1× longer than wide (oval bodies with elytra > 1.1× longer than wide in others). In addition, some genital characters are diagnostic including wider aedeagi (Fig. 9C) (slenderer aedeagi in *E. flavicornis* (Fig. 3C) and *E. jungchani* sp. nov. (Fig. 8C)) and slightly swollen spermathecal receptaculum (Fig. 9I) (swollen spermathecal receptaculum in *E. flavicornis* (Fig. 3C) and *E. houjayi* sp. nov. (Fig. 6C)).

###### Food plants.

Urticaceae: *Debregeasia orientalis* C. J. Chen; *Gonostegia hirta* (Blume) Miq.

###### Etymology.

This new species is named for Mei-Hua Tsou (曹美華), the first person to collect specimens.

###### Distribution.

Southern Taiwan. This species is restricted to the Hengchun Peninsula (Fig. 5).

### ﻿*Euphitrea nisotroides* species group

Adults of this group differ from those of the *E. flavicornis* group by the reddish brown prothorax and head, dark brown antenna except several basal antennomeres (the dark metallic bronze or blackish brown prothorax and head, and yellow antenna in *E. flavicornis* group), strongly swollen and symmetrical tarsi I in males (slightly swollen and asymmetrical tarsi I in males in *E. flavicornis* group), ventral surface of male aedeagus with membranous areas (ventral surface of male aedeagus without membranous areas in *E. flavicornis* group); and frontal ridge convex (frontal ridge flat in *E. flavicornis* group).

#### ﻿Included species

*Euphitrea nisotroides* (Chen, 1933) and *E. taiwana* Kimoto, 1991.

##### ﻿ Euphitrea nisotroides

Taxon classificationAnimaliaColeopteraChrysomelidae

(Chen, 1933)

D15F7742-5E37-549D-AB1C-D88E52AD497C

**Figure 10.**
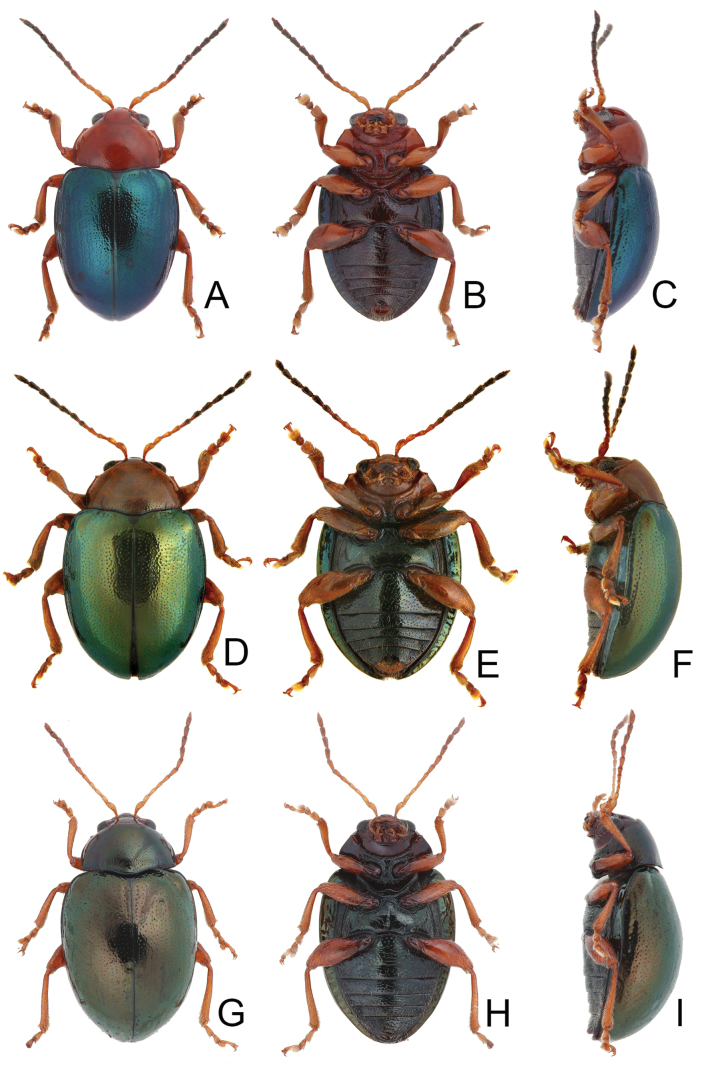
Habitus of *Euphitrea* species **A***E. nisotroides* Kimoto, male, dorsal view **B** ditto, ventral view **C** ditto, lateral view **D***E. taiwana* Kimoto, male, typical form, dorsal view **E** ditto, ventral view **F** ditto, lateral view **G***E. taiwana* Kimoto, female, color variation, dorsal view **H** ditto, ventral view **I** ditto, lateral view.

**Figure 11.**
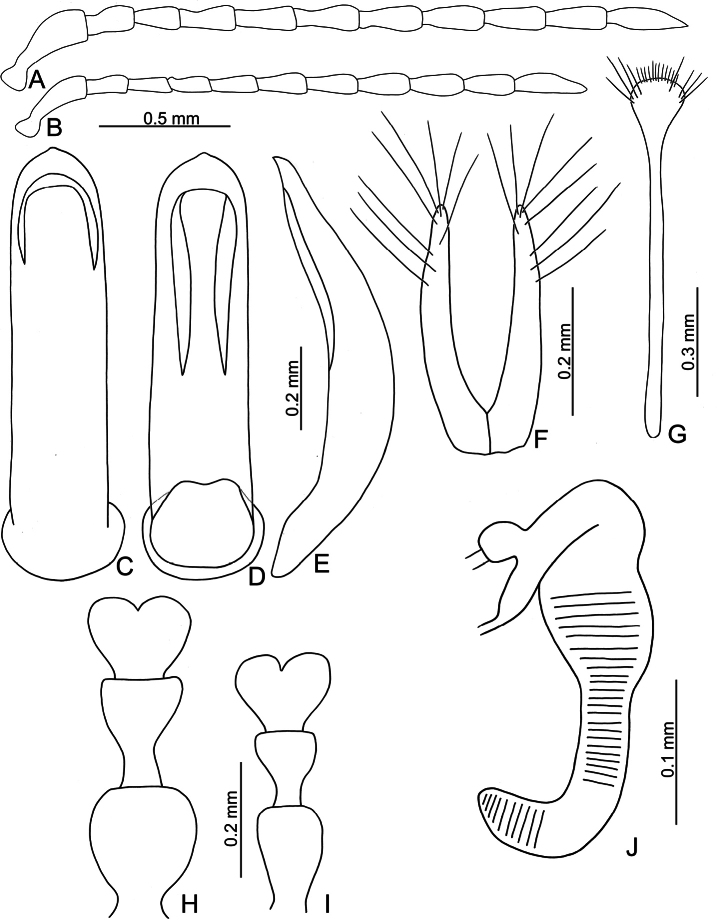
*Euphitrea nisotroides* (Chen) **A** antenna, male **B** antenna, female **C** aedeagus, dorsal view **D** aedeagus, ventral view **E** aedeagus, lateral view **F** gonocoxae **G** abdominal ventrite VIII, female **H** tarsus, male **I** tarsus, female **J** spermatheca.

**Figure 12.**
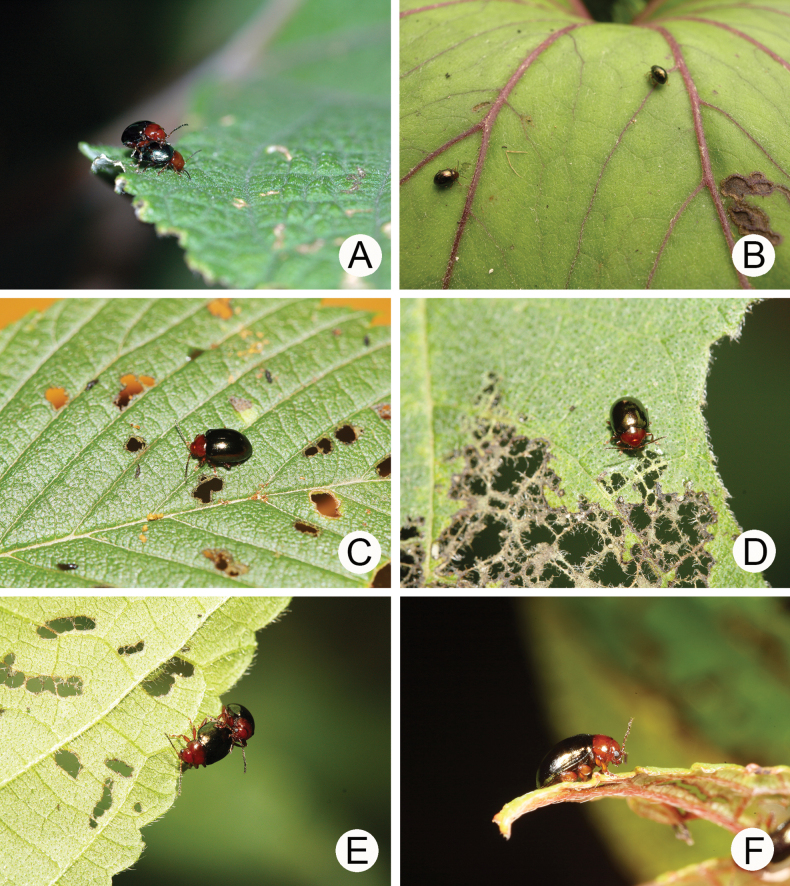
Field photographs of *Euphitrea* species **A** a couple of adults of *E. nisotroides* (Chen) on *Boehmeria nivea* (Urticaceae) **B** adults of *E. taiwana* Kimoto on *Petasites formosanus* (Asteraceae) **C** adult of *E. taiwana* Kimoto on *Ulmus uyematsui* (Ulmaceae) **D** adult of *E. taiwana* Kimoto on *Broussonetia monoica* (Moraceae) **E** a couple of adults of *E. taiwana* Kimoto on *Celtis formosana* (Cannabaceae) **F** adult of *E. taiwana* Kimoto on *Debregeasia orientalis* (Urticaceae).

**Figure 13.**
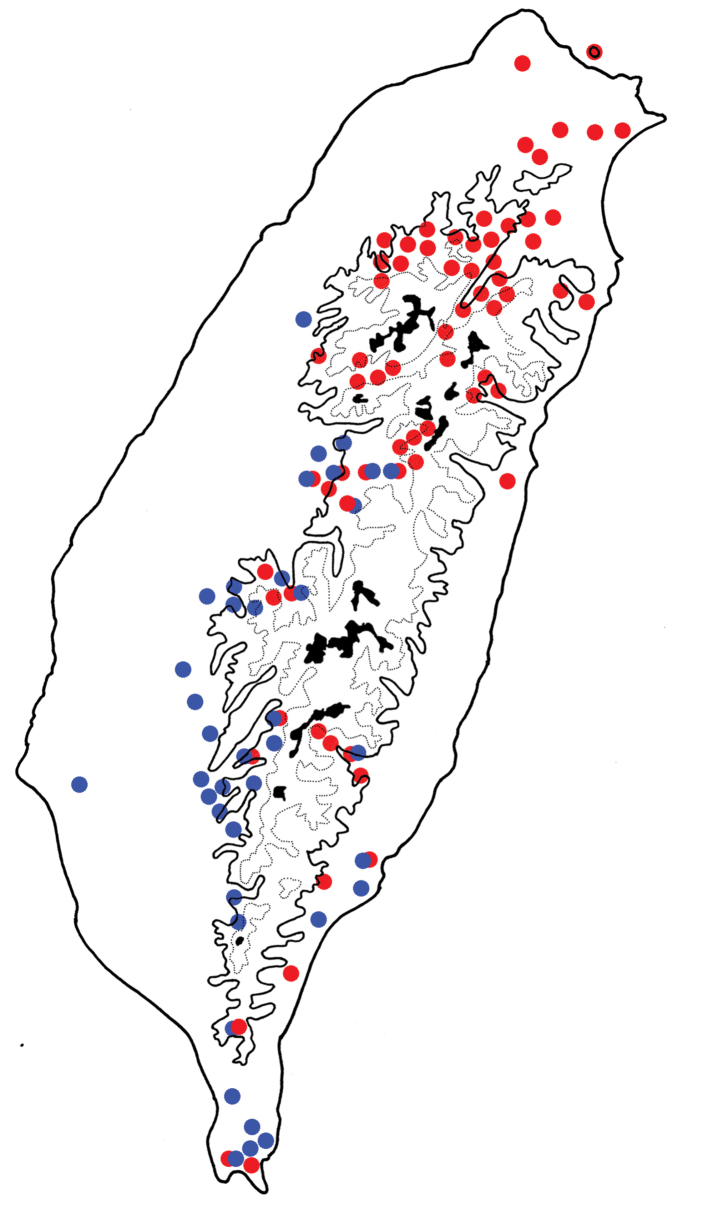
Distribution map of *Euphitrea flavicornis* species group in Taiwan; solid line 1000 m, broken line 2000 m, black areas 3000 m. Red dots *E. taiwana* Kimoto, blue dots *E. nisotroides* (Chen).

Figs 2E, 10A–C, 11, 12A, 13

Neorthaea nisotroides Chen, 1933: 92 (Taiwan: Taihorin); Chûjô 1935: 473 (part); Chûjô 1963: 399 (faunistic records); Chûjô 1958: 16 (Japan: North Borodino Island); Kimoto 1966: 35 (faunistic records); Kimoto 1970: 215 (faunistic records).
Euphitrea nisotroides : Kimoto, 1986: 59 (faunistic records); Kimoto, 1989: 263 (faunistic records); Kimoto 1991b: 19 (faunistic records); Yu et al. 1996: 251 (China: Fujian); Wang and Yang 2006: 211 (China: Gansu); Ruan and Yang 2017: 198 (China: Shaanxi); Bezděk and Konstantinov 2024: 509 (catalogue).

###### Type material examined.

***Lectotype*** • ♂ (SDEI, here designated to clarify its taxonomic status), labeled: “Taihorin (= Talin, 大林) / Formosa / H. Sauter, 1911 [p, w] // 7.VIII. [p, w] // Chen det [h, w] // Syntypus [p, r] // Coll. DEI / Eberawalde [p, w] // Neorthaea / nisotroides m. [h] / S. H. Chen det. [p, w] // SDEI Müncheberg / Col – 19931 [p, g]”. ***Paralectotypes*** • 1♂ (SDEI): “Taihorin (= Talin, 大林) / Formosa / H. Sauter, 1911 [p, w] // 7.VIII. [p, w] // Chen det [h, w] // Syntypus [p, r] // Coll. DEI / Eberawalde [p, w] // Euphitrea / nisotroides / (Chen, 1933) / det. Döberl 1999 [p, w] // SDEI Müncheberg / Col – 19930 [p, g]”.

###### Additional material examined.

**Taiwan. Chiayi** • 1♂, 1♀ (TARI), Arisan (= Alishan, 阿里山), 2–23.X.1918, leg. J. Sonan • 2♂, 3♀ (TARI), Lichia (里佳), 8.V.2019, leg. B.-X. Guo • 8♂, 2♀ (TARI), Laichi (來吉), 20.III.2009, leg. H. Lee • 2♂, 8♀ (TARI), Shihpanku (石盤谷), 15.V.2017, leg. B.-X. Guo • 1♂ (NHMUK), Tahorin (= Talin, 大林), III.1910, leg. S. G. Sauter; **Kaohsiung** • 10♂, 9♀ (TARI), Chiahsien (甲仙), 25.VI.2022, leg. Y.-F. Hsu • 1♀ (TARI), Chungchihkuan (中之關), 13.X.2012, leg. L.-P. Hsu • 1♂ (NHMUK), Hoozan (= Fengshan, 鳳山), III.1910, leg. H. Sauter • 1♂ (TARI), Hsiaokuanshan (小關山), 15.V.2016, leg. B.-X. Guo • 14♂, 20♀ (TARI), Kuotingshan (廓亭山), 26.VI.2022, leg. Y.-F. Hsu • 1♀ (KMNH), Liu Kui (= Liukuei, 六龜), 31.III.1986, leg. K. Baba • 1♂ (TARI), 27–28.VI.1981, leg. L. Y. Chou & C. H. Yang • 7♂, 4♀ (TARI), Meilungshan (美瓏山), 15.VI.2016, leg. B.-X. Guo • 1♂ (TARI), Shanping (扇平), 12.IV.2014, leg. W.-C. Laio • 1♀ (TARI), same but with “4.X.2014” • 1♂, 4♀ (TARI), Tengchih (藤枝), 2–5.VI.2008, leg. C.-F. Lee • 1♂ (KMNH), Tao Nah (= Tona, 多納), 30.V.1986, leg. K. Baba • 3♂, 2♀ (TARI), same locality, 28.III.2009, leg. C.-F. Lee • 3♂, 1♀ (TARI), same locality, 28.IX.2023, leg. Y.-T. Chung • 1♂, 1♀ (TARI), same but with “26.III.2024”; **Miaoli** • 1♀ (NHMUK), Daan River (大安溪) SW of Sanyi (三義), 24°21'30"N, 120°43'E, 150m, 26–28.X.2008, leg. L. Dembický; **Nantou** • 1♀ (TARI), Baibara (= Meiyuan, 眉原), 4–7.VII.1939, leg. Y. Miwa • 2♂ (TARI), Hokuzanko (= Peishankeng, 北山坑), 6.VII.1940, leg. M. Chujo • 1♀ (TARI), Hoshe (和社), 22.VII.1982, leg. L. Y. Chou & T. Lin • 1♂, 1♀ (TARI), Huisun Forest Recreation (惠蓀林場), 26.VIII.2013, leg. F.-S. Huang • 3♂, 2♀ (TARI), Kuantaoshan (關刀山), 5.VIII.2017, leg. B.-X. Guo • 43♂, 35♀ (NHMUK), 0.5km NW of Lushan (廬山), 121°10.876'N, 24°01.481'E, 1268 m, leg. H. Mendel, U. Ong, M. V. L. Barclay, R. Ewers • 1♂ (TARI), Nanshanchi (南山溪), 7.IV.2010, leg. Y.-T. Wang • 1♂ (TARI), same but with “10.VII.2010” • 2♂, 4♀ (TARI), Tungpu (東埔), 20–22.VI.1980, leg. C. C. Chen • 2♂, 6♀ (TARI), same locality, 25–29.IX.1980, leg. L. Y. Chou & T. Lin • 25♂, 26♀ (TARI), same locality, 28.IV.–2.V.1981, leg. T. Lin & C. J. Lee • 24♂, 24♀ (TARI), same locality, 5–8.X.1981, leg. T. Lin & W. S. Tang • 13♂, 6♀ (TARI), same locality, 18–23.XI.1981, leg. T. Lin & W. S. Tang • 47♂, 62♀ (TARI), same locality, 19–23.VII.1982, leg. L. Y. Chou & T. Lin • 3♂, 3♀ (TARI), same locality, 22–25.XI.1982, leg. K. C. Chou & S. P. Huang • 15♂, 20♀ (TARI), same locality, 20–24.VI.1983, leg. K. C. Chou & C. Y. Wong • 20♂, 48♀ (TARI), same locality, 16–20.IV.1984, leg. K. C. Chou & C. H. Yung • 34♂, 44♀ (TARI), same locality, 23–27.VII.1984, leg. K. C. Chou & C. H. Yang; 1♀ (TARI), Wanfengtsun (萬豐村), 11.VII.2007, leg. Y.-C. Chang • 12♂, 7♀ (TARI), same locality, 2.IV.2008, leg. W.-T. Liu • 1♂, 1♀ (TARI), Musha (= Wushe, 霧社), 18.V.–15.VI.1919, leg. T. Okuni • 1♂, 1♀ (TARI), same locality, 25.VI.–5.VII.1947, leg. Maa, Chen, & Lin • 1♂, 1♀ (TARI), same locality, 30.VIII.2.IX.1982, leg. L. Y. Chou & K. C. Chou • 2♂, 2♀ (TARI), same locality, 19–22.IV.1983, leg. K. C. Chou & S. P. Huang • 1♀ (TARI), same locality, 11–15.IX.1984, leg. K. S. Lin • 2♀ (TARI), 17.VIII.1984, leg. K. C. Chou • 1♂ (TARI), same locality, 11–15.IX.1984, leg. K. S. Lin • 1♂, 2♀ (TARI), 23.III.2009, leg. U. Ong • 1♂ (TARI), Yushih (幼獅), 4.VIII.1981, leg. T. Lin & W. S. Tang; **Pingtung** • 3♂, 6♀ (TARI), Chialeshui (佳樂水), 6.VII.2017, leg. B.-X. Guo • 1♂ (SDEI), Koshun (= Henchun, 恆春), VI.1912, leg. H. Sauter • 1♂, 1♀ (TARI), same locality, 25.IV.–25.V.1918, leg. J. Sonan • 1♀ (TARI), same locality, 4.IV.1040, leg. R. Matsuda • 1♂ (TARI), Lilungshan (里龍山), 5.XI.2009, leg. M.-H. Tsou • 1♀ (TARI), Manchou (滿州), 16–30.VIII.2009, leg. M.-L. Jeng • 2♂, 3♀ (TARI), Nanjenhu (南仁湖), 1.III.2011, leg. J.-C. Chen • 3♂, 2♀ (TARI), Nanjenshan (南仁山), 24.II.2009, leg. C.-F. Lee • 2♀ (TARI), Peitawushan (北大武山), 4.IV.2013, leg. Y.-T. Chung • 1♂ (TARI), same but with “12.IV.2013” • 6♂, 5♀ (TARI), same but with “22.IV.2014” • 1♂ (TARI), same but with “21.III.2015” • 1♀ (TARI), Tahanshan (大漢山), 18.VII.2007, leg. C.-F. Lee • 2♀ (TARI), Wutai (霧台), 11.IX.2023, leg. Y.-T. Chung; **Taichung** • 1♂, 2♀ (TARI), Chaipaotai (佳保台), 14–18.X.1980, leg. K. S. Lin & C. H. Wang • 2♂, 6♀ (TARI), Kukuan (谷關), 20–22.VI.1978, leg. K. S. Lin & K. C. Chou • 7♂, 5♀ (TARI), same locality, 14–17.X.1980, leg. K. S. Lin & C. H. Wang • 2♂ (TARI), same locality, 19.III.2014, leg. C.-F. Lee • 3♂, 2♀ (TARI), Pahsienshan (八仙山), 2.XI.2009, leg. M.-H. Tsou; **Tainan** • 1♂, 2♀ (TARI), Kantoushan (崁頭山), 1.V.2017, leg. W.-C. Liao; 1♀ (TARI), Kuantzuling (關子嶺), 30.IX.1965, leg. S. C. Chiu • 2♀ (TARI), Meiling (梅嶺), 28.XII.2008, leg. U. Ong; **Taitung** • 2♀ (TARI), Tipon (= Chihpen, 知本), 13.VI.1940, leg. M. Chujo • 1♀ (TARI), same locality, 17–18.II.1982, leg. L. Y. Chou & K. C. Chou • 3♂, 6♀ (TARI), same locality, 22.I.2013, leg. B.-X. Guo • 1♂, 1♀ (TARI), Motien (摩天), 5.X.2010, leg. C.-F. Lee • 1♂ (TARI), Rikiriki (= Lichi, 利吉), 21.III.1924, leg. N. Takeda • 4♂, 1♀ (1♂, 1♀: TARI; 3♂: NHMUK), Taito (= Taitung, 台東), 25.II.–27.III.1919, leg. S. Inamura, J. Sonan, M. Yoshino.

###### Redescription.

***Adults*.** Length 3.5–4.3 mm, width 2.1–2.5 mm (*n* = 772). Head, prothorax, and legs yellowish or reddish brown (Fig. 10A–C); meso- and metathoracic, and abdominal ventrites blackish brown; antennomeres I–IV yellowish brown, apical 1/2 of V dark brown, VI–XI black. Furrows lateral to vertex deep and long, apically extending beyond apical margins of eyes (Fig. 2E). Antennae (Fig. 11A) filiform in males, ratios of lengths of antennomeres I–XI 1.0: 0.5: 0.4: 0.5: 0.6: 0.6: 0.6: 0.6: 0.6: 0.6: 0.8; ratios of length to width from antennomeres I–XI 2.9: 1.9: 2.0: 2.3: 2.5: 2.5: 2.5: 2.6: 2.6: 2.7: 3.9; much smaller in females, ratios of lengths of antennomeres I–XI (Fig. 11B) 1.0: 0.5: 0.5: 0.5: 0.6: 0.6: 0.6: 0.6: 0.6: 0.6: 0.9; ratios of length to width from antennomeres I–XI 3.6: 2.3: 2.8: 2.6: 3.0: 2.4: 2.5: 2.4: 2.5: 2.5: 3.7. Pronotum 1.7× wider than long; disc shining, with sparse, fine punctures, moderately convex; lateral margins rounded; apical margins almost straight; basal margin slightly and medially convex. Elytra 1.3× longer than wide; disc with coarse punctures arranged longitudinally, with fine punctures between longitudinally arranged coarse punctures; lateral margins rounded, apically narrowed behind basal 1/3; humeral calli well developed, hind wings normal. Tarsomeres I of front and middle legs strongly swollen in males (Fig. 11H); less swollen and much smaller in females (Fig. 11I). Apical margin of abdominal ventrite V in males trilobed, notches shallow; apical margin of abdominal ventrite V broadly rounded in females. Aedeagus (Fig. 11C–E) slender, ~ 4.7× longer than wide; parallel-sided, apex widely rounded, slightly convex at middle of apical margin; moderately curved in lateral view; ventral surface with wide, longitudinal, sclerotized areas from apical 1/12 to middle, sides surrounded by membranous areas; tectum slightly sclerotized. Endophallic spiculae reduced. Gonocoxae (Fig. 11F) longitudinal and connected at base; each gonocoxa apically narrow; apex narrowly rounded or truncate; curved inwards; with eight long apical setae. Ventrite VIII (Fig. 11G) well sclerotized and small, short, and dense setae arranged into transverse line along apical margin, apical margin rounded, with four long setae at sides, spiculum extremely long. Spermathecal receptaculum (Fig. 11I) strongly swollen; pump long and curved; sclerotized spermathecal duct short beyond spermathecal gland, base of spermathecal gland enlarged and sclerotized.

###### Diagnosis.

**Figure 14.**
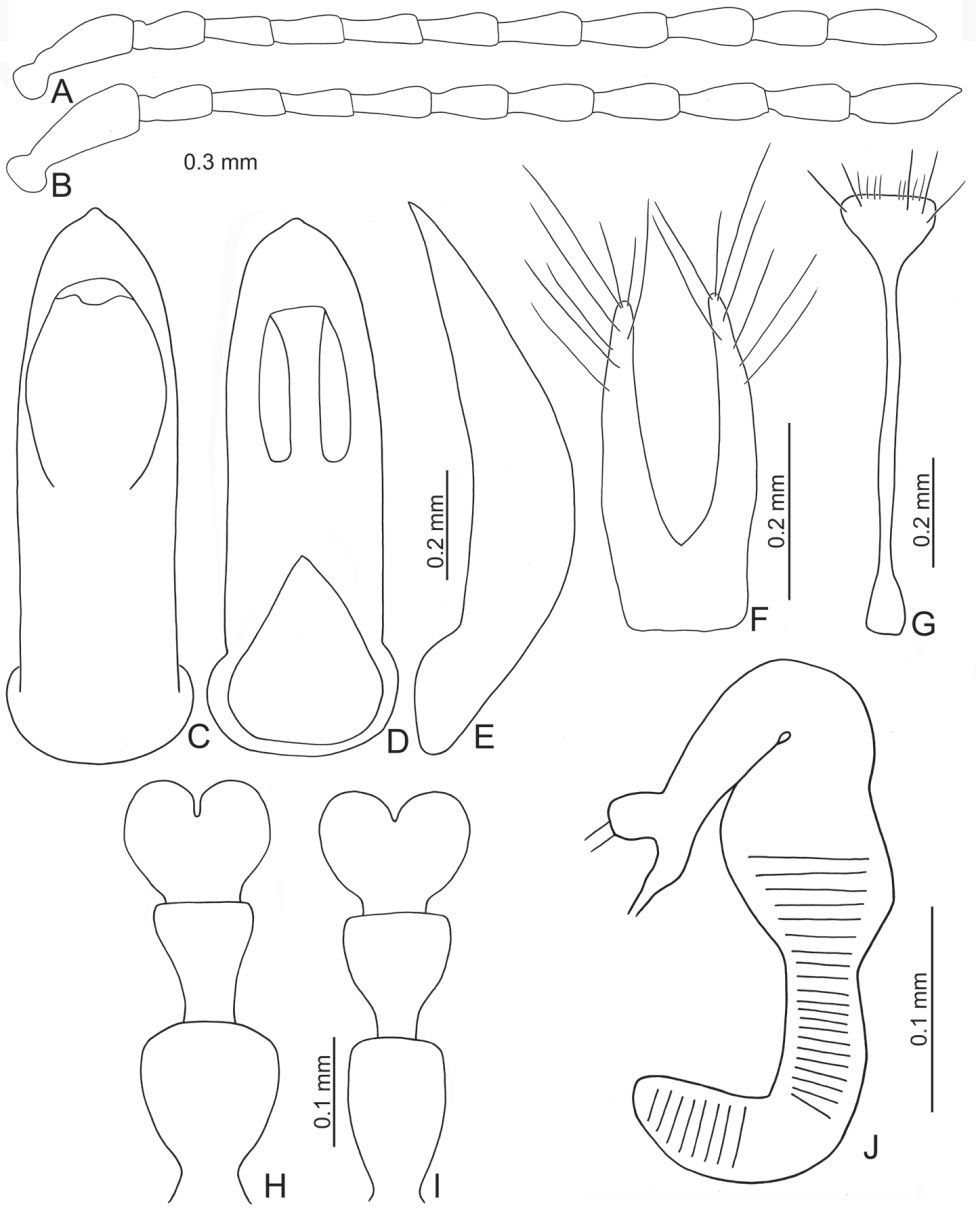
*Euphitrea taiwana* Kimoto **A** antenna, male **B** antenna, female **C** aedeagus, dorsal view **D** aedeagus, ventral view **E** aedeagus, lateral view **F** gonocoxae **G** abdominal ventrite VIII, female **H** tarsus, male **I** tarsus, female **J** spermatheca.

Adults of *Euphitrea nisotroides* are similar to those of *E. taiwana* with similar color patterns, but *E. nisotroides* can be easily recognized by its distinct, elongate longitudinal ridge on the vertex of the head (Fig. 2E) (short longitudinal ridges on the vertex in *E. taiwana*; Fig. 2F). In addition, the antennae and tarsi are sexually dimorphic (Fig. 11A, B, H, I), the aedeagi are slenderer (Fig. 11C, D) (4.7× longer than wide), and the apical margin of the abdominal ventrite VIII in females is rounded (Fig. 11G). These characters differ in *E. taiwana*, with similar sizes of antennae (Fig. 14A, B) and tarsi (Fig. 14H, I) in both sexes, wider aedeagi (Fig. C, D) (3.8× longer than wide) and truncate apical margin of abdominal ventrite VIII in females (Fig. 14G).

###### Food plants.

Cannabaceae: *Celtis sinensis* Pers.; Ulmaceae: *Zelkova serrata* (Thunb.) Makino; Urticaceae: *Boehmeria nivea* (L.) Gaudich. (Fig. 12A); *Debregeasia orientalis* C.J.Chen.

###### Distribution.

China, Japan, and Taiwan. Only one specimen was collected from the North Borodino Island, Japan (Chûjô 1958). No detailed studies was conducted on Chinese specimens. Records from China and Japan need further confirmation. The species is widespread in the lowlands of central and south Taiwan. Adults were not found higher than 2000 m elevation (Fig. 13).

##### ﻿ Euphitrea taiwana

Taxon classificationAnimaliaColeopteraChrysomelidae

Kimoto, 1991

6190F001-67EB-5102-BA9B-566AF19540AF

Figs 2F, 10D–I, 12B–F, 13, 14

Neorthaea nisotroides : Chûjô 1935: 473 (part); Chûjô 1965: 103 (faunistic records).
Euphitrea taiwana Kimoto, 1991a: 14 (Taiwan: Paling); Bezděk and Konstantinov 2024: 509 (catalogue).

###### Type material examined.

*Euphitrea taiwana* • 1♀ (KMNH): “ Pa Lin (巴陵), / Tao Yuan Hsien, / N-Taiwan / 14.VIII.1987 / Col. K. Baba [p, w] // Euphitrea / taiwana / Kimoto, n. sp. [h] / Det. S. Kimoto, 19[p]91 [h, w] // PARATOPOTYPE [p, b]” • 1♂ (KMNH): “ Pa Lon (巴陵?), / Tao Yuan Hsien, / N-Taiwan / 28.V.1989 / Col. K. Baba [p, w] // Euphitrea / taiwana / Kimoto, n. sp. [h] / Det. S. Kimoto, 19[p]91 [h, w] // PARATOPOTYPE [p, b]” • 1♂, 1♀ (KMNH): “Mei Fon (梅峰), / (Alt. 2350m) / M-Taiwan / 10.VI.1987 / Col. K. Baba [p, w] // PARATYPE [p, b] // Euphitrea / taiwana / Kimoto, n. sp. [h] / Det. S. Kimoto, 19[p]91 [h, w]”.

###### Additional material examined.

**Taiwan. Hsinchu** • 2♂ (TARI), Chenhsipao (鎮西堡), 11.VII.2014, leg. Y.-L. Lin • 10♂, 9♀ (TARI), Mt. Hinoki (= Kueishan, 檜山), 5.IV.1940, leg. M. Chujo • 1♂, 1♀ (TARI), Inoue (= Chingchuan, 清泉), 20.VII.1935, leg. M. Chujo • 1♂, 1♀ (TARI), Kuanwu (觀霧), 30.IV.2010, leg. C.-F. Lee • 5♂, 4♀ (TARI), Litungshan (李棟山), 23.III.2007, leg. M.-H. Tsou • 3♂ (TARI), same but with “6.VI.2010” • 1♀ (TARI), same locality, 27.X.2009, leg. S.-F. Yu • 1♀ (TARI), Lupi (魯壁), 10.III.2009, leg. S.-F. Yu • 2♂, 2♀ (TARI), Mamei (馬美), 25.IV.2008, leg. W.-T. Wu • 2♀ (TARI), same locality, 18.V.2008, leg. M.-H. Tsou • 2♂, 1♀ (TARI), Shiigao (= Maopu, 茅圃), 27–30.VI.1934, leg. M. Chujo • 2♂, 2♀ (1♂, 1♀: TARI; 1♂, 1♀: NHMUK), Shinchiku (= Hsinchu, 新竹), 1–30.VII.1918, leg. J. Sonan & K. Miyake • 1♂ (TARI), Sumakusu (司馬庫斯), 11.VII.2010, leg. M.-H. Tsou • 1♀ (TARI), Talu trail (大鹿林道), 25.III.2009, leg. Y.-L. Lin • 1♂ (TARI), Wufeng (五峰), 14–16.VII.1982, leg. K. C. Chou & C. C. Pan; **Hualien** • 1♂ (TARI), Hsinpaiyang (新白楊), 11.XII.2010, leg. W.-P. Chan • 1♂, 1♀ (TARI), Huitouwan (迴頭彎), 10.VII.2007, leg. C.-F. Lee • 1♂ (TARI), Karapao (卡拉寶), 14.VIII.1940, leg. M. Chujo • 2♂ (TARI), Karento (= Hualien, 花蓮), 20.VII.–4.VIII.1919, leg. T. Okuni • 1♂, 3♀ (TARI), Pilu (碧綠), 16.VI.2008, leg. C.-F. Lee; **Ilan** • 1♂ (TARI), Chilan (棲蘭), 17.III.2007, leg. M.-H. Tsou • 1♂ (TARI), Chiuchihtse (鳩之澤), 7.XII.2008, leg. M.-H. Tsou • 1♀ (TARI), Mt. Fanbao logging Rd. (飯包山林道), 1.IV.2014, leg. B.-C. Lai • 10♂, 11♀ (NHMUK), Fushan Botanical Park (福山植物園), 23–24.VII.2005, leg. A. C. Galsworthy • 1♂, 1♀ (TARI), same locality, 2.IV.2008, leg. M.-H. Tsou • 2♂, 2♀ (TARI), same but with “leg. H.-J. Chen” • 2♂ (TARI), same locality, 20.III.2009, leg. C.-F. Lee • 1♀ (TARI), Grian (= Ilan, 宜蘭), VI.–VII.1918, leg. Y. Yamazaki • 1♂ (TARI), Mingchi (明池), 25.V.2008, leg. M.-H. Tsou • 4♂, 8♀ (TARI), same locality; 2.VII.2008, leg. H.-J. Chen • 2♂, 8♀ (TARI), same locality, 20.IV.2017, leg. J.-C. Chen • 1♀ (TARI), Pailing (白嶺), 3.VII.2010, leg. M.-T. Tsou • 1♂, 1♀ (TARI), Shemi Lake (神祕湖), 23.X.2011, leg. C.-H. Hsieh • 1♀ (TARI), Sungluoshan (松蘿山), 4.VI.2017, leg. Y.-T. Wang • 2♂, 1♀ (TARI), Suyang (思源), 9.VI.2009, leg. S.-F. Yu • 3♂, 2♀ (TARI), same locality, 2.V.2012, leg. J.-C. Chen • 1♀ (TARI), Shikig(k)un (= Sunchi 四季), 22.V.1931, leg. R. Takahashi • 2♀ (TARI), same locality, 13.VIII.1933, leg. Y. Miwa • 1♂, 2♀ (TARI), 28.V.2009, leg. Y.-T. Wang • 3♂, 3♀ (TARI), same locality, 7.VII.2009, leg. H.-J. Chen • 3♀ (TARI), same but with “19.V.2010” • 1♂ (TARI), Sungluo Lake (松蘿湖), 3.IV.2009, leg. Y.-T. Wang • 1♂, 1♀ (TARI), Taiheizan (= Taipingshan, 太平山), 26–27.VIII.1923, leg. T. Shiraki • 1♀ (TARI), same locality, VII.1930, leg. S. Minowa • 1♀ (TARI), same locality, 10.VII.1940, leg. R. Matsuda • 2♀ (TARI), same locality, 17.VII.1940, leg. M. Chujo • 2♂, 3♀ (TARI), same locality, 19.VII.1940, leg. S. Miyamoto • 6♂, 5♀ (TARI), same locality, 26–28.VII.1983, leg. L. Y. Chou • 1♂, 2♀ (TARI), same locality, 7.IV.2007, leg. S.-S. Li • 1♀ (TARI), same locality, 25.IV.2007, leg. S.-F. Yu • 3♂, 4♀ (TARI), same but with “3.VI.2007” • 2♂, 6♀ (TARI), same but with “19.VI.2008” • 1♂, 4♀ (TARI), same locality, 8.VII.2008, leg. H.-J. Chen • 1♂, 1♀ (TARI), same locality, 20.IV.2015, leg. H. Lee • 1♂, 1♀ (TARI), Tuchang (土場), 1.III.2007, leg. S.-S. Li • 2♂, 1♀ (TARI), Yuanshan (員山), leg. 26.III.2009, leg. H.-J. Chen; **Kaohsiung** • 1♂ (TARI), Chungchihkuan (中之關), 16.IV.2012, leg. L.-P. Hsu • 1♂, 1♀ (TARI), Meilungshan (美瓏山), 15.VI.2016, leg. B.-X. Guo; **Keelung** • 5♂, 4♀ (TARI), Keelungyu Island (基隆嶼), 10.VI.2012, leg. C.-H. Hsieh; **Nantou** • 1♀ (TARI), Honbukei (= Penpuchi, 本部溪), 7.VII.1940, leg. M. Chujo • 6♂, 3♀ (KMNH), Hsitou (溪頭), 30–31.III.1980, leg. K. Sugiyama • 1♂, 1♀ (TARI), same locality, 17.III.2004, leg. H.-Y. Lee • 1♂, 1♀ (TARI), Kanetowan (加年端社), 15.IV.1910, leg. I. Nitobe • 1♀ (TARI), Kuantaoshan (關刀山), 5.VIII.2017, leg. B.-X. Guo • 2♀ (KMNH), Lushan (廬山), 6.VI.1976, leg. H. Makihara; 6♂, 2♀ (KMNH), same but with “20.VI.1976” • 1♂, 1♀ (KMNH), same locality, 3.VIII.1990, leg. S. Kimoto • 9♂, 7♀ (TARI), 23.III.2009, leg. U. Ong • 2♂, 4♀ (TARI), Meifeng (梅峰), 10.V.1979, leg. K. C. Chou • 2♂ (TARI), same locality, 20–22.VI.1979, leg. K. S. Lin & B. H. Chen • 1♀ (TARI), same locality, 18.VII.1979, leg. K. C. Chou • 1♂ (TARI), same locality, 22–29.VIII.1979, collectors unknown • 1♂ (TARI), same locality, 2–4.VI.1980, leg. L. Y. Chou & C. C. Chen • 1♂ (TARI), same locality, 8.VI.1980, leg. K. S. Lin & B. H. Chen • 2♂ (TARI), same locality, 26.VIII.1980, leg. K. S. Lin & C. H. Wang • 1♂ (TARI), same locality, 5–9.X.1980, leg. C. C. Chen & C. C. Chien • 5♂, 6♀ (TARI), same locality, 7–9.V.1981, leg. K. S. Lin & S. C. Lin • 1♀ (TARI), same locality, 24–26.VI.1981, leg. K. S. Lin & W. S. Tang • 2♂ (TARI), same locality, 28–29.VIII.1981, leg. L. Y. Chou & S. C. Lin • 2♂, 1♀ (TARI), same locality, 22.V.1982, leg. L. Y. Chou • 14♂, 7♀ (TARI), same locality, 15.VII.1982, leg. S. C. Lin & C. N. Lin • 1♂, 4♀ (TARI), same locality, 31.VIII.–2.IX.1982, leg. L. Y. Chou & K. C. Chou • 2♂, 2♀ (TARI), same locality, 4–7.X.1982, leg. K. C. Chou • 8♂, 3♀ (TARI), same locality, 19–21.IV.1983, leg. K. C. Chou & S. P. Huang • 2♂ (TARI), same locality, 30.VII.1983, leg. L. Y. Chou • 1♂ (TARI), same locality, 8–11.V.1984, leg. K. C. Chou & C. C. Pan • 1♂, 2♀ (TARI), same locality, 20–21.VI.2009, Y.-T. Wang • 1♀ (TARI), Nanshanchi (南山溪), 7.IV.2010, leg. Y.-T. Wang • 1♂ (TARI), same locality, 3.VII.2010, leg. T.-Y. Liu • 2♂ (NHMUK), Rueiyan River Major Wildlife Habitat (瑞岩溪野生動物重要棲息環境), 24°05'848"N, 121°10'859"E, 2096m, leg. H. Mendel & M. V. L. Marclay • 5♂, 8♀ (TARI), Shalihsien trail (沙里仙林道), 9–12.VI.2013, leg. Y.-T. Wang • 2♂, 3♀ (TARI), Sungkang (松崗), 6.VIII.1984, leg. K. S. Lin • 1♀ (TARI), same locality, 15–19.VIII.1984, leg. K. C. Chou • 4♂, 2♀ (TARI), same locality, 13–15.IX.1984, leg. K. S. Lin & S. C. Lin • 1♂ (TARI), 10.IV.2016, leg. Y.-T. Chung • 1♀ (TARI), same locality, 2.VI.2016, leg. B.-X. Guo • 2♂, 1♀ (TARI), Tsuifeng (翠峰), 23.V.1982, leg. L. Y. Chou • 1♂, 2♀ (TARI), Tungpu (東埔), 20–22.VI.1980, leg. C. C. Chen • 1♂, 1♀ (TARI), same locality, 25–29.IX.1980, leg. L. Y. Chou & T. Lin • 10♂, 24♀ (TARI), same locality, 28.IV.–2.V.1981, leg. T. Lin & C. J. Lee • 2♂, 3♀ (TARI), same locality, 5–8.X.1981, leg. T. Lin & W. S. Tang • 3♀ (TARI), same locality, 18–23.XI.1981, leg. T. Lin & W. S. Tang • 45♂, 44♀ (TARI), same locality, 19–23.VII.1982, leg. L. Y. Chou & T. Lin • 1♂, 2♀ (TARI), same locality, 22–25.XI.1982, leg. K. C. Chou & S. P. Huang • 3♂, 2♀ (TARI), same locality, 20–24.VI.1983, leg. K. C. Chou & C. Y. Wong • 4♂, 10♀ (TARI), same locality, 16–20.IV.1984, leg. K. C. Chou & C. H. Yung • 4♂, 9♀ (TARI), same locality, 23–27.VII.1984, leg. K. C. Chou & C. H. Yang • 5♂, 4♀ (TARI), Tunyan (屯原), 6.VII.2012, leg. Y.-F. Hsu • 1♂, 1♀ (TARI), same locality, 14.V.2013, leg. B.-X. Guo • 1♀ (TARI), Tzuchungshan (自忠山), 7.VI.2013, leg. Y.-T. Wang • 1♂, 1♀ (TARI), Wanfengtsun (萬豐村), 13.IV.2010, W.-T. Liu • 1♂ (TARI), same locality, 15.IV.2010, leg. Y.-C. Chang • 1♂, 1♀ (TARI), Musha (= Wushe, 霧社), 18.V.–15.VI.1919, leg. T. Okuni, J. Sonan, K. Miyake, M. Yoshino • 4♂, 9♀ (TARI), same locality, 25.VI.–5.VII.1947, leg. Maa, Chen, & Lin • 1♂, 1♀ (TARI), 23–28.VI.1981, leg. K. S. Lin & W. S. Tang • 26♂, 21♀ (TARI), same locality, 30.VIII.–2.IX.1982, leg. L. Y. Chou & K. C. Chou • 2♀ (TARI), same locality, 7–8.X.1982, leg. K. C. Chou • 48♂, 49♀ (TARI), same locality, 19–22.IV.1983, leg. K. C. Chou & S. P. Huang • 4♂, 4♀ (TARI), same locality, 7.V.1984, leg. K. C. Chou & C. C. Pan • 3♂ (TARI), same locality, 4.VIII.1984, leg. K. S. Lin • 3♂, 2♀ (TARI), same locality, 17.VIII.1984, leg. K. C. Chou • 1♂ (TARI), same locality, 23.III.2009, leg. U. Ong • 1♀ (TARI), Yushih (幼獅), 4.VIII.1981, leg. T. Lin & W. S. Tang; **Pingtung** • 1♂ (TARI), Kenting (墾丁), 24–28.VI.1981, leg. T. Lin & C. C. Pan • 1♀ (TARI), Koshun (= Henchun, 恆春), 4.IV.1040, leg. R. Matsuda • 3♀ (TARI), Tahanshan (大漢山), 18.VII.2007, leg. C.-F. Lee • 1♀ (TARI), same but with “leg. M.-H. Tsou” • 1♀ (TARI), same locality, 20.VII.2007, leg. M.-H. Tsou; **Taichung** • 1♀ (TARI), Anmashan (鞍馬山), 21.IV.2010, leg. C.-F. Lee • 2♂, 3♀ (TARI), same but with “19.X.2011” • 16♂, 16♀ (TARI), Chiapaotai (佳保台), 14–18.X.1980, leg. K. S. Lin & C. H. Wang • 1♀ (TARI), Hassenzan (= Pahsienshan, 八仙山), 6.III.1930, leg. J. Sonan • 2♂ (KMNH), Kukuan (谷關), 9.VI.1976, leg. H. Makihara • 2♂, 1♀ (TARI), same locality, 20–22.VI.1978, leg. K. S. Lin & K. C. Chou • 8♂, 6♀ (TARI), same locality, 14–17.X.1980, leg. K. S. Lin & C. H. Wang • 1♂ (TARI), Pilu (畢祿), 1.VII.2008, leg. M.-H. Tsou • 1♀ (TARI), Tahsuehshan (大雪山), 1.V.2012, W.-T. Liu • 3♀ (TARI), Wuling (武陵), 27–29.VI.1979, leg. K. S. Lin & L. Y. Chou • 11♂, 8♀ (TARI), Wushihkeng (烏石坑), 19.III.2008, leg. C.-F. Lee • 1♂, 1♀ (TARI), same but with “13.VII.2008”; **Taipei** • 1♀ (TARI), Rimogan (= Fushan, 福山), 5.IV.1940, leg. M. Chujo • 1♀ (TARI), same locality, 22.VI.2008, leg. H.-J. Chen • 3♂, 2♀ (TARI), same but with “11.V.2009” • 3♂, 3♀ (TARI), Habun (= Hayao, 哈呅; near Fushan), 12.V.1933, leg. M. Chujo • 1♂ (TARI), Kanko (乾溝), 18.IX.1924, leg. T. Shiraki • 1♂ (TARI), Pinglin (坪林), 10.II.2009, leg. H. Lee • 5♂, 2♀ (TARI), Rahau (= Hsinhsien, 信賢), 13.V.1933, leg. M. Chujo • 4♀ (TARI), Urai (= Wulai, 烏來), 28.III.1932, leg. M. Chujo • 2♂ (TARI), same but with “15.VI.1932” • 2♂, 3♀ (TARI), same but with “4.IV.1940” • 1♂, 1♀ (KMNH), same locality, 27.VII.1990, leg. S. Kimoto • 4♂, 3♀ (TARI), same locality, 27.III.2007, leg. Y.-F. Yu; 1♀ (NHMUK), same locality, 18.V.2007, leg. G. Martin & D. L. J. Quicke • 1♂ (TARI), same locality, 4.III.2008, leg. H.-J. Chen • 1♀ (TARI), Sozan (= Yangmingshan, 陽明山), 12.IX.1938, leg. M. Chujo • 4♀ (TARI), same locality, 19.IV.2007, leg. C.-F. Lee • 1♀ (TARI), Yingtzuling (鶯子嶺), 6.VII.2015, leg. S.-P. Wu; **Taitung** • 2♀ (TARI), Tipon (= Chihpen, 知本), 13.VI.1940, leg. M. Chujo • 2♀ (TARI), Hsiangyang (向陽), 23.VI.2010, leg. M.-H. Tsou • 1♀ (TARI), same locality, 28.III.2014, leg. W.-C. Huang • 1♀ (TARI), Lichia (利嘉), 19.V.2009, leg. U. Ong • 1♀ (TARI), same locality, 15.VII.2014, leg. Y.-T. Chung • 1♀ (TARI), same locality, 16.VII.2014, leg. Y.-T. Wang • 1♀ (TARI), Liyuan (栗園), 23.VI.2010, leg. M.-H. Tsou • 1♀ (TARI), same locality, 19.VI.2013, leg. C.-F. Lee • 1♂ (TARI), same but with “24.VII.2013” • 1♂, 1♀ (TARI), same locality, 14.III.2014, leg. W.-C. Huang • 2♂ (TARI), same but with “28.III.2014” • 1♀ (TARI), Motien (摩天), 23.V.2011, leg. C.-F. Lee • 1♀ (TARI), Rikiriki (= Lichi, 利吉), 21.III.1924, leg. N. Takeda • 1♀ (NHMUK), Taihanroku (= Taipan, 台板), 8–18.IV.1908, leg. H. Sauter • 1♀ (TARI), Wulu (霧鹿), 29.III.2011, leg. C.-F. Lee; **Taoyuan** • 3♀ (TARI), Lalashan (拉拉山), 22.VII.2008, leg. H.-J. Chen • 5♂, 6♀ (TARI), same locality, 8.III.2009, leg. S.-F. Yu • 1♂, 1♀ (TARI), Hsuanyuan (萱源), 16.III.2008, leg. M.-H. Tsou • 3♂, 6♀ (TARI), Paling (巴陵), 3–5.V.1983, leg. K. C. Chou & C. C. Pan • 1♂ (TARI), same locality, 19.IV.2009, leg. S.-F. Yu • 1♀ (TARI), same locality, 21.III.2010, leg. M.-H. Tsou • 1♂ (TARI), Suleng (四稜), 17.III.2007, leg. M.-H. Tsou.

###### Redescription.

***Adults.*** Length 2.8–3.8 mm, width 1.7–2.3 mm (*n* = 928). Head, prothorax, and legs yellowish or reddish brown (Fig. 10D–F); meso- and metathoracic, and abdominal ventrites blackish brown; antennomeres I–IV yellowish brown, apical 1/2 of V dark brown, VI–XI black. Furrows lateral to vertex shallow and short, apically reaching or slightly exceeding apical margins of eyes (Fig. 2F). Antennae (Fig. 14A) filiform in males, ratios of lengths of antennomeres I–XI 1.0: 0.5: 0.5: 0.5: 0.6: 0.6: 0.7: 0.7: 0.7: 0.7: 0.8; ratios of length to width from antennomeres I–XI 3.1: 2.0: 2.2: 2.4: 2.7: 2.3: 2.5: 2.5: 2.2: 2.4: 3.1; similar in females, ratios of lengths of antennomeres I–XI (Fig. 14B) 1.0: 0.5: 0.5: 0.6: 0.6: 0.6: 0.6: 0.6: 0.6: 0.6: 0.8; ratios of length to width from antennomeres I–XI 2.6: 2.1: 2.5: 2.8: 2.5: 2.3: 2.3: 2.3: 2.4: 2.2: 3.1. Pronotum 1.7× wider than long; disc shining, with sparse, fine punctures, moderately convex; lateral margins rounded; apical margins almost straight; basal margin slightly and medially convex. Elytra 1.3× longer than wide; disc with coarse punctures arranged longitudinally, with fine punctures between longitudinally arranged coarse punctures; lateral margins rounded, apically narrowed behind basal 1/3; humeral calli well developed, hind wings normal. Tarsomeres I of front and middle legs strongly swollen in males (Fig. 14H); less swollen in females (Fig. 14I). Apical margin of abdominal ventrite V in males trilobed, notches shallow; apical margin of abdominal ventrite V broadly rounded in females. Aedeagus (Fig. 14C–E) wide, ~ 3.8× longer than wide; parallel-sided, apex widely rounded, slightly convex at middle of apical margin; moderately curved in lateral view; ventral surface with wide, longitudinal, sclerotized areas from apical 1/6 to middle, sides surrounding with membranous areas; tectum membranous. Endophallic spiculae reduced. Gonocoxae (Fig. 14F) longitudinal and connected at base; each gonocoxa apically narrow; apex narrowly rounded or truncate; curved inwards; with eight long apical setae. Ventrite VIII (Fig. 14G) well sclerotized and small, short, and less dense setae arranged into transverse line along apical margin, apical margin truncate, with two or three long setae at sides, spiculum extremely long. Spermathecal receptaculum (Fig. 14J) strongly swollen; pump long and curved; sclerotized spermathecal duct short beyond spermathecal gland, base of spermathecal gland enlarged and sclerotized.

###### Color variation.

Most individuals collected from Ilan county have dark reddish brown heads and prothoraces and yellowish brown antennae and legs (Fig. 10G–I), which look like members of *E. flavicornis* species group.

###### Diagnosis.

Adults of *Euphitrea taiwana* are similar to those of *E. nisotroides* with similar color patterns, but *E. taiwana* can be easily recognized by the shorter longitudinal ridges on the vertex of the head (Fig. 2F) (long longitudinal ridges on vertex in *E. nisotroides* (Fig. 2E)). In addition, similar sizes of antenna (Fig. 14A, B) and tarsi (Fig. 14H, I) are present in both sexes, the aedeagi are comparatively wider (Fig. 14C, D) (3.8× longer than wide) and the apical margin of abdominal ventrite VIII is truncate in females (Fig. 14G). These characters differ from those of *E. nisotroides*, which possess sexually dimorphic antennae (Fig. 11A, B) and tarsi (Fig. 11H, I) in both sexes, more slender aedeagi (Fig. 11C, D) (4.7× longer than wide) and rounded apical margin of abdominal ventrite VIII in females (Fig. 11G).

###### Food plants.

Asteraceae: *Petasites formosanus* Kitam. (Fig. 12B); Cannabaceae: *Celtis formosana* Hayata (Fig. 12E); Lythraceae: *Lagerstroemia subcostata* Koehne; Moraceae: *Broussonetia monoica* Hance (Fig. 12D); Rosaceae: *Prunus campanulata* Maxim.; Ulmaceae: *Ulmus uyematsui* Hayata (Fig. 12C); Urticaceae: *Debregeasia orientalis* C. J. Chen (Fig. 12F); *Gonostegia hirta* (Blume) Miq.; *Oreocnide pedunculata* (Shirai) Masam.

###### Distribution.

This species is extremely common in Taiwan, including a small island, Keelungyu Island (基隆嶼), near northern Taiwan (Fig. 13). They are rather dominant in northern Taiwan. Some populations occur higher than 2000 m elevations.

### ﻿Key to Taiwanese species of *Euphitrea*

**Table:** 

1	General color dark metallic bronze but antennae and legs yellow, tarsomeres I in males slightly swollen and asymmetrical	***E. flavicornis* group (2)**
–	General color metallic green and blue but head, prothorax, and legs reddish brown, antennae dark brown except several basal antennomeres, tarsomeres I in males strongly swollen and symmetrical	***E. nisotroides* group (5)**
2	Humeral calli well developed, hind wings normal (Fig. 4A)	**3**
–	Humeral calli and hind wings reduced (Fig. 4B)	***E. houjayi* sp. nov.**
3	Body shape oval, 1.2–1.4× longer than wide (Figs 1A–C; 7A–C)	**4**
–	Body shape transversely oval, 1.1× longer than wide (Fig. 7D–F)	***E. tsoui* sp. nov.**
4	Apices of aedeagi straight in lateral view (Fig. 3D), tectum membranous (Fig. 3C)	***E. flavicornis* Chen**
–	Apices of aedeagi curve in lateral view (Fig. 8D), tectum sclerotized (Fig. 8C)	***E. jungchani* sp. nov.**
5	Longitudinal ridges on the vertex of heads distinct and long (Fig. 2E)	***E. nisotroides* Chen**
–	Longitudinal ridges on the vertex of heads indistinct and short (Fig. 2F)	***E. taiwana* Kimoto**

## ﻿Discussion

In adults of Chinese species, the external morphology is rather diverse, including genital characters of the aedeagi, gonocoxae, and abdominal ventrites VIII in females (Zhang and Yang 2006). By contrast, genital characters of Taiwanese species are of limited diagnostic value, although other external characters are reliable for species identities. It will be good to see if this study can be supported or not based on molecular data in the future.

Adults of *Euphitrea* are commonly collected because they are polyphagous and diurnal. Thus, they are collected easily by sweeping and trapping so large numbers of specimens are available for study. This allows thorough delimitation of species distribution, diversity, and discovery of color variations. Modern taxonomic studies benefit from such comprehensive sampling efforts.

## Supplementary Material

XML Treatment for Euphitrea flavicornis

XML Treatment for Euphitrea houjayi

XML Treatment for Euphitrea jungchani

XML Treatment for Euphitrea tsoui

XML Treatment for Euphitrea nisotroides

XML Treatment for Euphitrea taiwana

## ﻿Additional information

### Author ORCIDs

Chi-Feng Lee https://orcid.org/0000-0003-1996-0557

### Data availability

All of the data that support the findings of this study are available in the main text.
